# New York State Climate Impacts Assessment Chapter 04: Buildings

**DOI:** 10.1111/nyas.15200

**Published:** 2024-12-09

**Authors:** Nicholas B. Rajkovich, Carrie Brown, Illya Azaroff, Erik Backus, Shannon Clarke, Jared Enriquez, Bethany Greenaway, Meghan T. Holtan, Jamal Lewis, Ozgem Ornektekin, Laurie Schoeman, Amanda Stevens

**Affiliations:** ^1^ School of Architecture and Planning University at Buffalo Buffalo New York USA; ^2^ Resource Refocus, LLC Oakland California USA; ^3^ Department of Architecture Technology New York City College of Technology and +LAB Architect Brooklyn New York USA; ^4^ Department of Civil and Environmental Engineering Clarkson University Potsdam New York USA; ^5^ New York State Division of Homeland Security and Emergency Services Albany New York USA; ^6^ Department of Geography and Planning University at Albany Albany New York USA; ^7^ Urban and Regional Planning University at Buffalo Buffalo New York USA; ^8^ Rewiring America Washington District of Columbia USA; ^9^ Ko2 Consulting New York New York USA; ^10^ Council on Environmental Quality Executive Office of the President Washington District of Columbia USA; ^11^ New York State Energy Research and Development Authority Albany New York USA

**Keywords:** adaptation, buildings, climate change, impacts, infrastructure, New York State, resilience, vulnerability

## Abstract

New York State has nearly 5.3 million buildings, and all of them are vulnerable in some way to the impacts of climate change. Understanding these impacts is critical, because risks to buildings not only threaten individual lives but also pose threats to community‐level resilience. This chapter examines the impacts of climate change on buildings and, by extension, the people and communities they shelter and support. It also highlights building types and populations that are at particular risk and presents adaptation strategies to protect the state's existing and future building stock from climate impacts.

## 1 TECHNICAL WORKGROUP KEY FINDINGS

New York State has nearly 5.3 million buildings, and all of them are vulnerable in some way to the impacts of climate change. Understanding these impacts is critical, because risks to buildings not only threaten individual lives but also pose threats to community‐level resilience. This chapter examines the impacts of climate change on buildings and, by extension, the people and communities they shelter and support. It also highlights building types and populations that are at particular risk and presents adaptation strategies to protect the state's existing and future building stock from climate impacts.

**Key Finding 1: Buildings of all ages, functions, and locations across New York State are vulnerable to the impacts of climate change**. Each of the state's 12 assessment regions will experience a range of impacts, including more severe storms, coastal and inland flooding, and increasing temperatures, all of which can affect building structures and systems, operations, and occupants. Local and regional factors, including geography, zoning, and socioeconomic disparities, also affect vulnerability and will shape site‐specific adaptations.

**Key Finding 2: Given the long lifespan of buildings, new construction and retrofits that consider long‐term climate projections will better address future climate risk**. New York State's new and existing buildings are expected to support many generations of use, while individual components of those buildings may be replaced more frequently. As climate change risks increase throughout this century, design decisions that align buildings’ and building components’ life cycles with future climate projections and expected hazards will lead to more cost‐effective, sustainable, and future‐ready buildings.

**Key Finding 3: Climate impacts to buildings can ripple to many different parts of a community**. Buildings are integrated and interdependent with other sectors, including agriculture, energy, transportation, and health services. Damage to buildings can disrupt these interdependent systems and compound the direct impacts of building loss and harm to individuals and communities. Addressing these cross‐sector impacts will require not only resilient building design, but also a multidisciplinary approach and insight into interconnected sectors to improve community resilience.

**Key Finding 4: Communities of color, Tribal communities, and low‐income communities are more likely to congregate, live, and work in buildings that have greater exposure to climate hazards**. In addition, people who are very young or very old, as well as those experiencing physical or developmental disabilities, are more vulnerable to building failures. Additional resources and policies will be required to respond to the disproportionate impacts of climate change in these communities.

**Key Finding 5: Individual adaptation and resilience strategies can address multiple climate impacts**. Buildings in New York State will face various climate hazards over their useful lives. Strategies such as green roofs, for example, can address both flooding and the urban heat island effect, while also providing cobenefits like reducing cooling load. Resilient design strategies can be implemented in many types of buildings and can increase community‐level resilience to climate change.

BOX 1 **Developments since the 2011 ClimAID assessment**

The 2011 ClimAID assessment did not include a chapter specifically dedicated to the buildings sector. Starting in 2014, the University at Buffalo, L&S Energy Services, and Weather Analytics worked with the New York State Energy Research and Development Authority (NYSERDA) to produce a suite of documents assessing the impacts of climate change on buildings and the built environment.^1^ The authors of the current assessment reviewed these documents, updated their findings as needed, and incorporated the results into this chapter.

The previous suite of documents did not discuss climate justice in the built environment, nor did they discuss the relationship among hazards, impacts, vulnerable populations and systems, and adaptation and resilience strategies. Additionally, the attribution of extreme weather events to climate change has advanced since those documents were published. This chapter attempts to close these knowledge gaps by:

• Updating the hazards data with newer projections from Columbia University.

• Discussing equity and climate justice specifically in the context of buildings.

• Examining the links among hazards in conjunction with their impacts on building occupants and communities.

## 2 INTRODUCTION AND BACKGROUND

Buildings support the social infrastructure in which New Yorkers live, learn, and work, and they host essential services like health care and education. This chapter reviews the best available information sources to quantify what is known about impacts of climate change on New York State's buildings sector observed to date, impacts projected in the decades ahead, and opportunities to adapt and build resilience. It cites and assesses evidence from technical literature; analyzes data from state and federal agencies and other sources; and integrates perspectives from practitioners who design, build, and operate buildings in New York.

The background material in this section characterizes the scope and context of the state's buildings sector; summarizes key climate hazards and nonclimate stressors that interact with these hazards; introduces considerations related to equity, climate justice, and Indigenous communities in New York State; and touches upon some key opportunities for positive change. Subsequent sections assess the state of knowledge on climate impacts and adaptation as follows:

- **Section** **3** discusses observed and projected impacts to the buildings sector, building occupants, and communities for climate hazards expected in New York State.
- **Section** **4** describes communities and building types that are at risk.
- **Section** **5** outlines key adaptation and resilience strategies.
- **Section** **6** takes a deeper look at opportunities for positive change that can grow out of climate adaptation efforts, and it identifies emerging topics and research needs. This section also provides a conclusion, summarizing the major findings and recommendations presented in the chapter.
- The Traceable Accounts appendix examines each key finding in depth. It provides citations that support each assertion, and it presents the authors’ assessment of confidence in each finding.
- **Case studies** highlight examples of climate change impacts on buildings in New York, along with adaptation and resilience strategies that could serve as models for others. These case studies are not included in the chapter proper but are available through links provided in the chapter.

### 2.1 Sector scope and context

The Federal Emergency Management Agency (FEMA) estimates that there are nearly 5.3 million buildings in New York State, containing nearly 14 billion square feet of space.^2^, ^3^ These buildings have a collective value of over $2 trillion.^2^ New York's building stock continues to grow. For instance, Class A office space in new and existing buildings in New York City alone increased by 45 million square feet between 2000 and 2017.^4^

Although the New York City and Long Island regions account for only a small percentage of the state's total land area, the density of buildings in New York City reaches more than 15,000 buildings per square mile (Figure 4-1).^3^ Many rural counties have a density of fewer than 90 buildings per square mile.^3^ Table 4-1 presents a summary of the total number, square footage, and value of buildings in each of the regions used for this assessment. Figure 4-2 shows the percentage of the state's buildings in each region.

**FIGURE 4-1:**
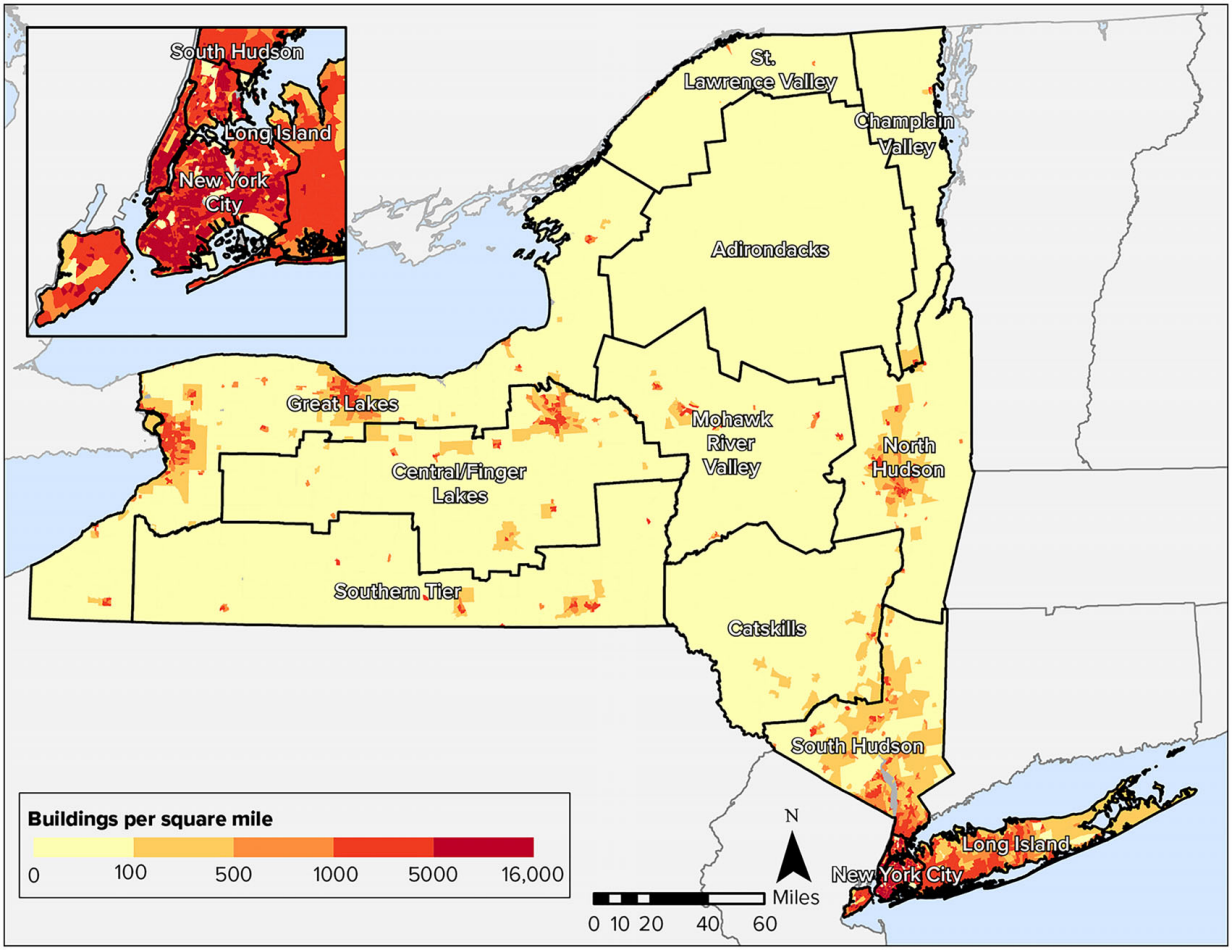
Building density in New York State. Data from Ray et al.^3^

**TABLE 4-1:** Summary of New York State's building stock by assessment region.

Region	Number of buildings	% of New York State building stock	Total square footage (thousand sq. feet)	Total square footage as % of state building stock	Total (full replacement value) ($)	Total replacement value as % of state building stock	Total (content) ($)	Total value of contents as % of state building stock
Adirondacks	69,961	1.33	111,484	0.81	$15,541,092.29	0.57	$9,290,609.70	0.53
Catskills	181,404	3.44	332,445	2.41	$56,778,808.00	2.10	$35,232,780.00	2.00
Central/Finger Lakes	403,366	7.64	848,112	6.16	$132,245,672.22	4.89	$89,203,990.34	5.07
Champlain Valley	70,942	1.34	135,691	0.98	$20,146,980.51	0.74	$13,188,826.10	0.75
Great Lakes	953,301	18.06	2,016,597	14.64	$322,493,266.78	11.92	$215,698,007.66	12.25
Long Island	1,001,307	18.97	2,204,007	16.00	$510,998,166.00	18.88	$326,556,756.00	18.55
Mohawk River Valley	206,800	3.92	396,244	2.88	$57,241,742.55	2.12	$37,314,032.56	2.12
New York City	1,099,707	20.84	4,838,303	35.12	$1,058,949,002.00	39.13	$688,028,754.00	39.09
North Hudson	367,071	6.95	819,254	5.95	$135,998,702.00	5.03	$90,946,220.00	5.17
South Hudson	598,514	11.34	1,468,710	10.66	$309,895,224.00	11.45	$197,136,950.00	11.20
Southern Tier	270,200	5.12	507,587	3.68	$72,579,314.00	2.68	$48,925,450.00	2.78
St. Lawrence Valley	55,243	1.05	98,009	0.71	$13,456,641.65	0.50	$8,742,005.64	0.50
**Total **	**5,277,816**	**100**	**13,776,441**	**100**	**$2,706,324,612.00**	**100**	**$1,760,264,382.00**	**100**

*Note*: Data from FEMA (n.d.).^2^

**FIGURE 4-2:**
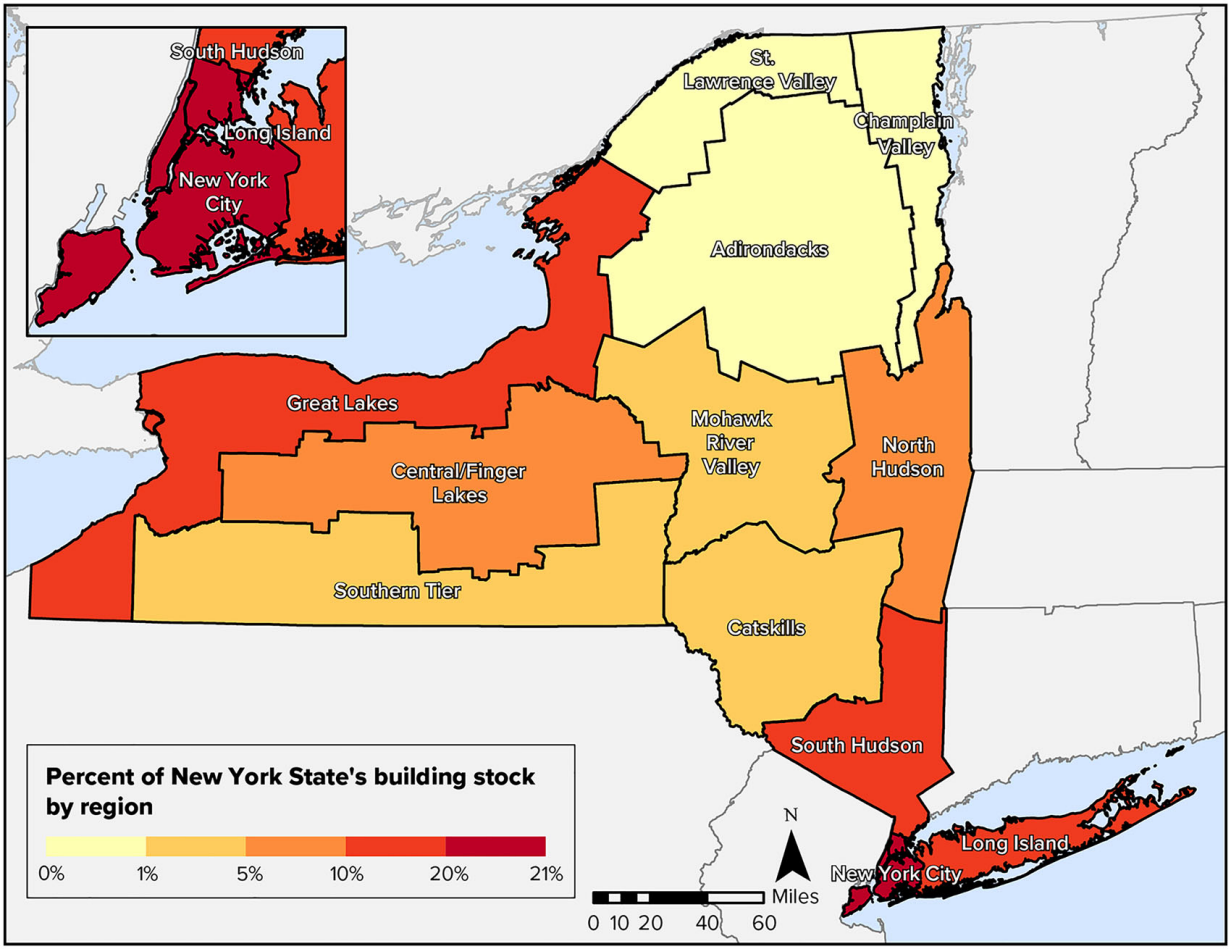
Regional distribution of New York State's building stock. Data from FEMA (n.d.).^2^

As shown in Table 4-2, about 90% of the state's buildings are residential (76% single family, 14% multifamily); 7% are for commercial use, recreation, and entertainment; and 2% are industrial. Buildings used for agriculture, community services, and public services each make up less than 1% of the state's buildings.^2^

**TABLE 4-2:** Number and square footage of buildings in New York State, by type.

Property type classification codes (New York State Department of Taxation and Finance)	Building types included (FEMA HAZUS)	Number of buildings	Percentage of total building count	Total square footage (thousand sq. feet)	Percentage of total statewide square footage
100: Agricultural	Agriculture facilities and offices	20,485	0.39%	58,027	0.42%
200: Residential	Single‐family dwellings, manufactured homes	4,023,230	76.23%	5,936,953	43.09%
	Multifamily homes, temporary lodging, dormitories, nursing homes	730,870	13.85%	4,340,394	31.51%
400: Commercial	Retail, wholesale trade, personal and repair services, personal and technical services, banks, parking garages	298,749	5.66%	2,136,083	15.51%
A500: Recreation and entertainment	Entertainment, recreation, theaters	57,801	1.10%	218,318	1.58%
600: Community services	Schools, school administration offices, colleges and universities, churches, nonprofit organizations	43,327	0.82%	385,845	2.8%
700: Industrial	Heavy industrial, light industrial, food/drugs/chemicals, metal and minerals processing, high technology, construction facilities and offices	90,544	1.72%	600,999	4.36%
800: Public services	General government services, emergency response	12,810	0.24%	99,821	0.72%
Total		5,277,816	100%	13,776,441	100%

*Note*: The table shows variations in how organizations designate building types. The New York State Department of Taxation and Finance uses a property type classification code, shown in column 1, to classify buildings in New York State for assessment purposes. FEMA's Hazards United States Multi‐Hazard (HAZUS‐MH) tool provides building data across sectors of the economy (as shown in column 2). This table applies FEMA data to the New York State Department of Taxation and Finance's building classification system to characterize the state's building stock. Data from FEMA (n.d.).^2^

Approximately 31% of New York's building stock was built in 1939 or earlier; 57% was built between 1940 and 1999; and 12% was built since 2000 (refer to Figure 4-3).^5^

**FIGURE 4-3:**
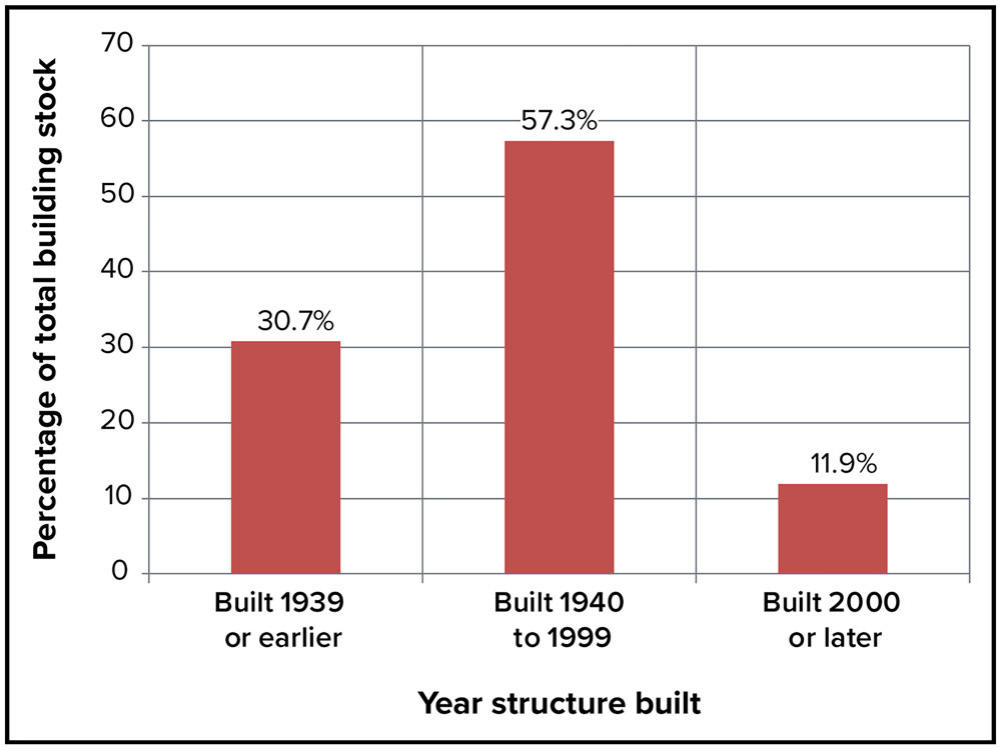
Percentage of total building stock by age of construction in New York State, 2021. Data from U.S. Census Bureau (2021).^5^

### 2.2 Key climate hazards

The FEMA National Risk Index identifies 18 natural hazards that affect communities in the United States.^6^ These hazards are used as the basis of county and state‐level hazard mitigation plans, such as the New York State Hazard Mitigation Plan. For this assessment, the Technical Workgroup conducted a literature review to examine the hazards identified in the FEMA National Risk Index to determine if they are applicable to New York State, and if the hazard can be attributable to climate change. Table 4-3 lists the 14 hazards that meet these criteria; summarizes the damage, probability, and the number of events expected to occur each year; and notes where in the chapter the hazard is discussed in further detail.

**TABLE 4-3:** Climate‐related hazards in the FEMA National Risk Index applicable to New York State.

Hazard as defined in the FEMA National Risk Index	Annualized loss (1996–2018)^7^	Daily probability in New York State^7^	Daily probability of a severe event in New York State^7^	Annual number of events^7^	Annual number of severe events^7^	Attribution of trends to human‐induced climate change^8^, ^9^	Location in chapter
Heat wave	$86 K	0.93%	0.00%	3	0	Increase in average temperatures, heat wave frequency and intensity	3.1.1: Increasing temperatures
Snowstorm	$15.5 M	20.44%	0.27%	72	0	Increase in average temperatures, decrease in cold wave frequency and intensity, heavier precipitation totals	3.1.3: Changes in precipitation
Ice storm	$4.07 M	0.53%	0.23%	1	0		
Cold wave	$855 K	2.84%	0.00%	9	0		
Hurricane	$758 K	0.05%	0.04%	0	0	Increase in rain rates	3.1.4: Extreme events
Coastal hazards	$3.75 M	1.32%	0.10%	3	0	Seiches are associated with hurricanes and severe storms	3.1.2: Sea level rise, coastal flooding, and seiches
Tsunami/seiche	$22 K	0.13%	0.00%	0	0		
Flooding	$129.9 M	21.75%	2.39%	77	8	Increase in the frequency, intensity, and amount of heavy precipitation	3.1.3: Changes in precipitation
Wind	$21.4 M	41.65%	0.22%	149	0	Severe convective storms may be more frequent and have increased rain rates	3.1.4: Extreme events
Tornado	$6.37 M	3.00%	0.13%	8	0		
Hail	$3.67 M	13.60%	0.00%	48	0		
Lightning	$466 K	4.62%	0.00%	16	0		
Landslide	$20 K	0.04%	0.00%	0	0	Not addressed by these sources	
Wildfire	$8720	0.09%	0.00%	0	0	Increase in fire weather expected with climate change	3.1.4: Extreme events

*Note*: Data from New York State Division of Homeland Security and Emergency Services (2019)^7^ and Seneviratne et al.^8^

Three additional hazards not listed in the FEMA National Risk Index were added to this assessment because of their connections to climate change: sea level rise and subsidence, indoor air quality, and pests.

The economic impact of climate change on the building stock of New York State is challenging to model. Most studies of the impact of climate change on the building stock (e.g., Ray et al.^3^) use the damage from past events as a proxy for the damage that could occur in the future with climate change. This chapter's estimates of damage to the building stock follow this approach.

### 2.3 Nonclimate factors

Stressors in other sectors will have a direct impact on buildings and the built environment. For example, habitat and biodiversity loss has a reciprocal relationship with buildings. Construction of the built environment often results in the modification or even destruction of natural systems. Climate change will add stress to these systems, in some cases causing them to shift or fail over time. Conversely, natural systems, such as wetlands, provide ecosystem services to the built environment, such as drainage and flood protection. Support from ecosystems is a critical component of a resilient built environment.^10^

There is an inherent interdependence among a community's buildings and its electrical, communications, water, and transportation infrastructure (refer to Section 3.3 for more discussion of cascading and cross‐sector impacts). Understanding the interconnected and synergistic relationship between the regional infrastructure grid and local buildings is becoming more critical as hazards and risks continue to grow. For example, in the energy sector, higher temperatures and heat waves will cause changes in peak load profiles and grid stability, which will affect building operations. Several building decarbonization studies have identified the challenges of moving to all‐electric systems as the climate warms.^11^ For buildings to be adapted to current and future conditions, there must be a continued and strategic engagement with—and connection to—regional planning and capital development processes, as well as continual training of building occupants and facility managers.

Building stock vulnerability differs across the state because of regional and local socioeconomic disparities. These disparities impact how buildings can be adapted to a changing climate. Rising insurance and reinsurance costs along the coast or in mapped floodplains could make it undesirable in certain locations to maintain the existing building stock and pursue development in the long term. Over time, more locations in the state could find themselves in regulatory flood zones as the official maps incorporate sea level rise and the increasing extent, severity, and frequency of inland flood events.

Relatively well‐resourced communities may be able to relocate to locations less at risk from climate change impacts. Such relocations could displace existing populations and cause climate gentrification (refer to Section 6.2).^12^ When rising insurance costs affect low‐income or frontline communities, home and building owners in these neighborhoods may not be able to afford to rebuild following a natural disaster. With the loss of these structures, there is a corresponding loss to the tax base that supports basic government services.

The geographic distribution of hazards, zoning, and codes also affects the ability of a region to adapt. For example, rural regions have a lower concentration of construction firms and construction employees relative to urban and suburban areas and could, therefore, have less capacity to modify existing buildings, repair them after extreme events, or construct new buildings designed for resilience to future climate hazards.^3^

### 2.4 Equity and climate justice

Climate change vulnerabilities intersect with and are exacerbated by underlying and systemic social and economic stressors and fragilities, as discussed in the Assessment Introduction. Low‐income communities, as well as Indigenous Peoples and Black and Hispanic populations, are more vulnerable to climate change impacts due to other forms of social and economic disadvantage and marginalization, including legacies of displacement, historical and ongoing racial and ethnic discrimination, lack of access to resources, and higher exposure to environmental pollutants. For similar reasons, other socially marginalized groups are also more vulnerable to climate change. These include unhoused, formerly and currently incarcerated, LGBTQ+, and immigrant populations, particularly those of low and moderate income, as the Society and Economy chapter explores in more depth.

Buildings preserve and improve human life by providing protection against the natural environment. Resources like insulation improve the ability of a building to protect its occupants from extreme weather and wildlife. However, because of high levels of income inequality in New York State, access to these resources differs among racial, ethnic, rural, and urban groups. In some instances, these differential vulnerabilities are rooted in legacies of discrimination associated with policy decisions, disinvestment, and actions such as redlining of urban neighborhoods to limit or prevent access to credit in areas where large concentrations of Black residents and other ethnic minorities lived. For example, green infrastructure is unevenly distributed in New York City; low‐income neighborhoods and communities of color have less access to climate‐regulating ecosystem services compared to neighborhoods with predominantly white and high‐income residents.^13^, ^14^ In addition, historical land dispossession and forced displacement of Indigenous Peoples contributes to these groups’ climate vulnerabilities.^15^

Centering equity in adaptation, resilience, and mitigation actions and strategies is critical for New York State to respond successfully and sustainably to climate change. Indigenous, local, and place‐based information are important sources of climate change knowledge, especially when collaboratively incorporated into adaptation and resilience responses. The buildings sector and every community in the state have the potential to contribute to climate solutions that reduce vulnerabilities, enhance resilience, and foster just transitions.

#### 2.4.1 Climate injustice in the buildings sector

To assess climate impacts on buildings across New York State, it is important to consider the disproportionate impacts experienced by vulnerable populations across the state and the implications of these disparities when developing adaptation and resilience strategies and policies. In the built environment, climate injustice is caused by four interconnected factors:

- Wealthier Americans have carbon footprints that substantially exceed those of low‐income populations,^16^ yet low‐income communities often shoulder a disproportionate share of the consequences and have less capacity to adapt.
- Low‐ to moderate‐income populations spend a large proportion of their income on energy because of limited incomes, inefficient household energy appliances, and older and substandard homes.^17^ These factors lead to higher energy consumption for basic tasks, resulting in elevated energy bills. It is, therefore, important to replace these appliances with energy‐efficient alternatives. However, the transition to such appliances often requires a substantial financial investment, making it unaffordable for low‐income households. Consequently, these households remain burdened with disproportionately high energy bills, perpetuating a cycle of energy injustice and exacerbating the challenges faced by these communities.
- The impacts of climate change are not evenly distributed across the building stock and population. Some buildings have greater exposure to natural hazards (e.g., located in a floodplain where land is less expensive). Older or poorly maintained buildings may be more vulnerable to climate‐driven weather events (e.g., high winds during a hurricane). In addition, a legacy of racial segregation (e.g., redlining) and other forms of socioeconomic marginalization have forced vulnerable populations into these higher‐risk geographies and lower‐quality buildings, increasing their exposure to climate‐related hazards.^18^, ^19^
- Those who have benefited from the use of fossil fuels and those who are most harmed by climate change are often segregated from each other by time, social stratification, and physical distance. Examples include people outside of a given jurisdictional boundary (e.g., pollution from power plants in the Midwest causing acid rain in the Adirondacks) and those inside a boundary but who are not recognized (e.g., children in K–12 schools not yet eligible to vote).

#### 2.4.2 Climate justice in housing

A lack of investment in the production and maintenance of affordable housing, as well as discriminatory land use, environmental, and housing policies, has led to disinvestment in and deterioration of the existing housing stock and made historically marginalized communities more vulnerable to the impacts of climate change.^13^, ^20^ Structurally compromised units are often occupied by communities of color and clustered together in low‐income neighborhoods. These correlations were driven by racially discriminatory policies, such as redlining, that manipulated market forces and concentrated wealth and investment in whiter and higher‐income neighborhoods. Over decades, residents of whiter neighborhoods benefitted from access to capital and policies that enabled them to purchase new, well‐built homes or properly maintain and upgrade their existing buildings, making them less vulnerable to climate‐related impacts.^13^ Conversely, Black and Hispanic residents, Indigenous Peoples, and other residents of color were denied access to wealth and wealth‐building opportunities. They were barred from purchasing homes in wealthier neighborhoods, denied credit for purchase and upgrades, and forced to congregate in racially segregated, often high‐poverty neighborhoods. These communities are often sited in areas at elevated risk from the effects of extreme weather and other climate impacts, thus increasing the susceptibility of these communities’ homes and other buildings, and the built environment in general, to climate impacts. Within these communities, older adults, children, and those with preexisting medical conditions face additional risks from climate impacts.

Counties, cities, towns, villages, school districts, and special districts use property taxes to fund schools, police and fire departments, roads, and other public services and infrastructure that can help communities adapt to climate change. With less disposable income and wealth on average compared to the general population, the underserved groups described above have lower tax bases in their communities. This disadvantage results in fewer resources available to these communities to invest in services like education and further undermines the resilience of these communities across the state.

### 2.5 Indigenous communities

As noted in the Assessment Introduction, nine federally recognized or state‐recognized Tribal Nations are located in New York, and the state has nearly 400,000 residents with American Indian or Alaska Native heritage.^21^, ^22^ Land reduction and forced migration led to contemporary conditions in which Tribal lands across the United States experience increased exposure to climate change risks and hazards and diminished economic value.^15^ Tribal governments are responding by preparing homes and other buildings for a climate‐changed future. Additionally, Tribal renewable energy projects and architecture are demonstrating the principles of resilient design to local and national audiences. For example, in their 2019 *Climate Vulnerability Assessment*, the Shinnecock Nation details how sea level rise affects buildings through both inundation of houses and saltwater intrusion into home wells.^23^ The 2013 *Climate Change Adaptation Plan for Akwesasne* in the Saint Regis Mohawk Reservation identifies the impact of climate change on indoor air quality in homes.^24^ The plan also details building‐related resilience and mitigation strategies, including removing brush and woody debris from around houses, cutting trees close to power lines and homes to prevent storm damage, developing green building programs, and encouraging residents to remove items around the home that could collect standing water after storms to reduce insect‐borne disease.^24^

Other Tribal Nations in New York State are incorporating sustainable design principles into their buildings. For example, in the Central/Finger Lakes region, the Tsha’ Thoñswatha’ fire station built by the Onondaga Nation in 2015 (Figure 4-4) uses locally sourced wood, incorporates daylighting, and relies on solar power and a geothermal heat pump for in‐floor heating. The net‐zero energy building also serves as a community hall that can seat approximately 150 people and has a community kitchen. Onondaga Council member Tadodaho Sid Hill summarized, “We have a building that is designed to work with Nature and to be energy self‐sufficient.”^25^ The Onondaga Nation has also installed a solar array “in the shape of the Hiawatha Belt, demonstrating beauty in form and function.”^26^ The Seneca Nation is investing in renewable energy, including installing LED lighting in all Tribal buildings and a solar‐powered microgrid at their Allegany fish hatchery. They are currently conducting a feasibility study for a solar‐powered microgrid to power the Tribal administration building, community center, and wastewater treatment plant during emergencies.^27^ In 2019, the Oneida Nation implemented energy efficiency upgrades in 27 Nation‐owned buildings.

**FIGURE 4-4:**
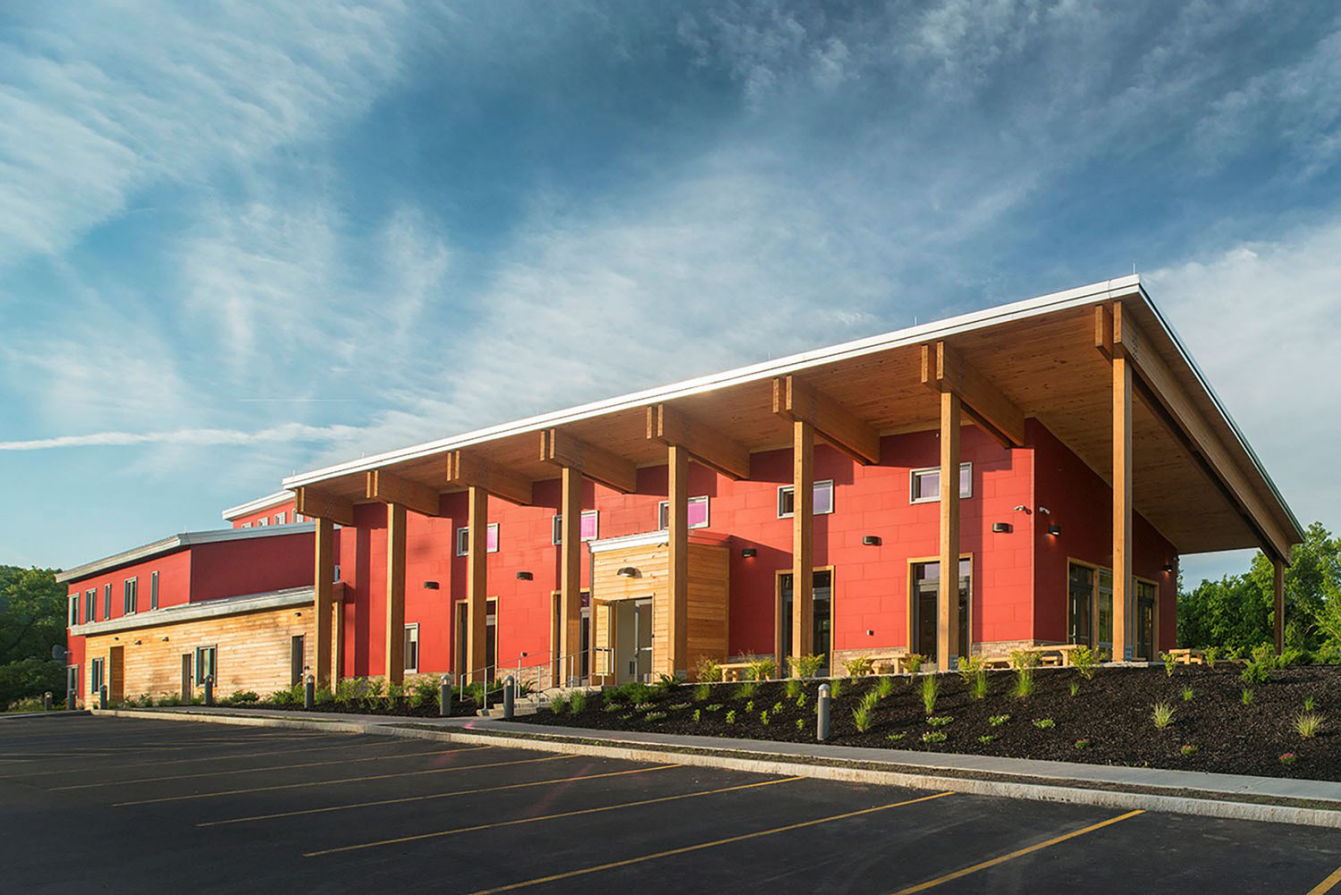
The net‐zero Tsha’ Thoñswatha’ fire station of the Onondaga Nation. Photo copyright © John Griebsch, 2023.

### 2.6 Opportunities for positive change

Climate change is expected to lead to primarily negative direct outcomes for New York State's buildings, aside from reducing winter heating needs as temperatures increase. By rising to the challenge and adapting to climate hazards, however, the state has opportunities to create additional positive outcomes. For example, better buildings can reduce energy burdens for residents and create safer, healthier spaces in which to live, work, and learn. Building projects can also embrace the concept of regenerative design, which involves creating spaces that reconnect occupants with nature while improving the condition of the surrounding environment.^28^, ^29^ Moreover, the state has opportunities to advance sustainability and resilience at the same time, as many strategies to increase resilience also reduce energy use and greenhouse gas emissions, and vice‐versa. Synergies and cobenefits of resilient buildings are explored throughout this chapter and discussed in further detail in Section 6.

## 3 OBSERVED AND PROJECTED IMPACTS

No location or building type is immune to the impacts of climate change. Every county across the state is at risk from climate‐driven hazards already, and vulnerability will increase over the next century. Many of the climate‐driven impacts to buildings and the built environment are caused by gradual changes in air temperature and associated precipitation patterns,^30^ which change average conditions as well as extremes (refer to Section 5 for adaptation and resilience strategies). In New York State, heat waves will become increasingly common over the next century as average temperatures increase.^31^ Because warming increases evaporation and puts more moisture into the air, the number of heavy rainstorms is likely to increase.^31^ In the Great Lakes region, warming water temperatures and reduced ice cover on Lake Erie and Lake Ontario may compound the issue by causing heavier lake‐effect rain and snow events,^31^ which can lead to building damage through flooding or roof collapse.

As the air temperature increases, over time, the need for heating buildings will decrease and the need for cooling will increase.^32^ Observed data on heating degree days and cooling degree days over the past several decades already reflect this trend.^33^ While these trends may decrease greenhouse gas emissions associated with boilers and furnaces, rising temperatures will increase electrical peaks in the summer associated with cooling.^34^ New York State is also anticipating shifting to a winter‐peaking load as heating systems are electrified.^35^ These gradual changes in temperature and coincident wet bulb (humidity) conditions could also make passive ventilation (i.e., providing ventilation without the use of mechanical systems) more challenging because this strategy works best in drier climates with cooler nights.

The gradual shift in average air temperatures and precipitation patterns is unlikely to cause direct damage to buildings and building structures.^36^, ^37^ However, extreme events are likely to cause damage to structures. Overall, climate‐related impacts are expected to shorten the lifespan of building materials.^38^ Figure 4-5 summarizes the effects of climate change on buildings and building occupants. Section 3.1 discusses each of these hazards and their impacts on buildings and their components.

**FIGURE 4-5:**
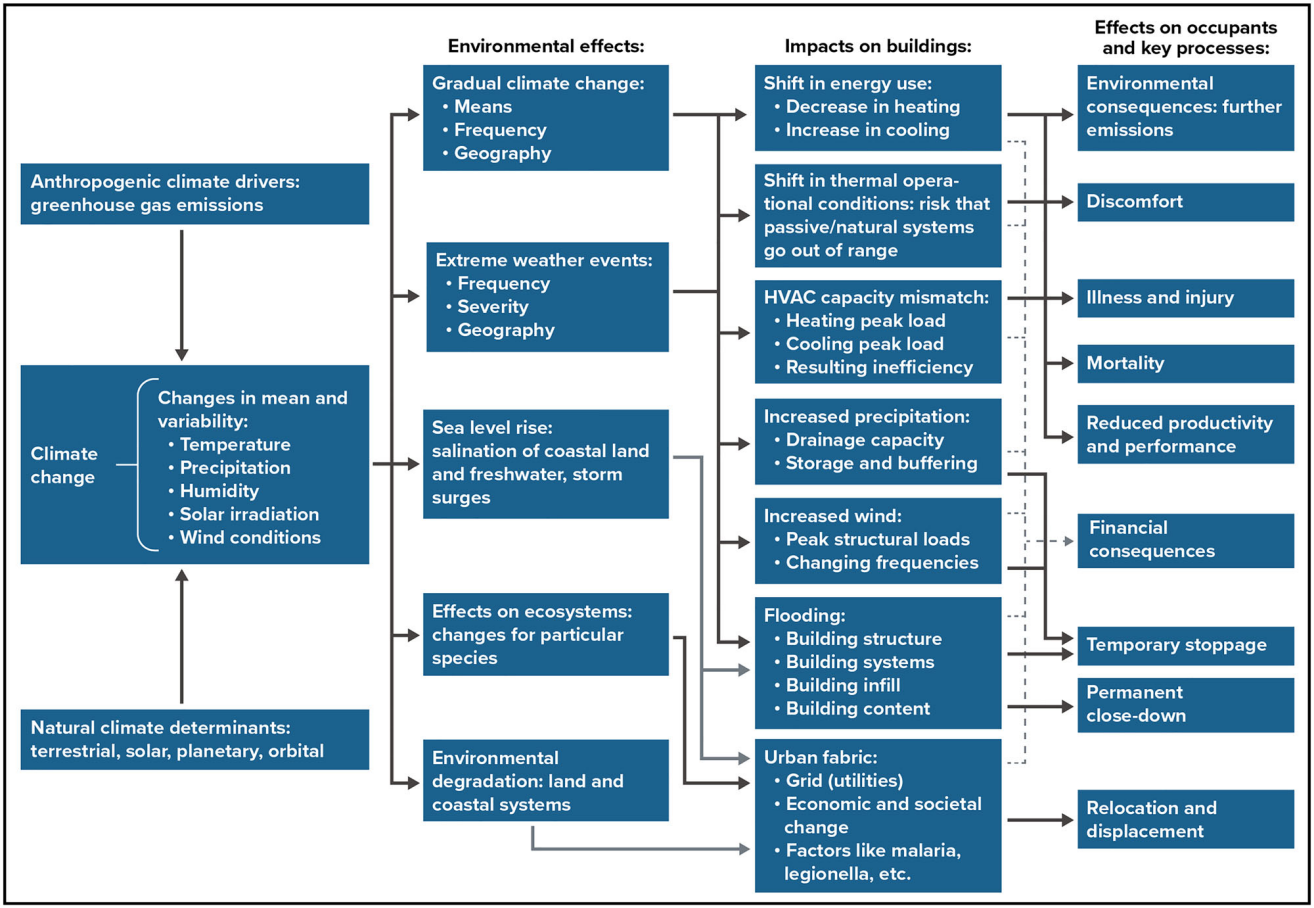
Climate change impacts on buildings and effects on building occupants. Figure by de Wilde and Coley.^39^

### 3.1 Climate‐driven impacts

#### 3.1.1 Increasing temperatures

From 1901 to 2022, average temperatures in New York State increased by almost 2.6°F, and the warmest 10‐year periods in recorded history have occurred since 2000.^31^ All seasons have experienced warming throughout the state.^31^ Annual average temperatures in New York State are projected to continue to rise due to climate change, and multiday extreme heat waves are expected to increase in frequency and duration.^31^ Urban heat islands compound the effects of heat waves on buildings as pavements gradually release stored heat at night, which can produce higher nighttime temperatures.^40^

- **Observed and projected impacts to buildings**. All buildings in New York State are vulnerable to increasing temperatures, though the severity and extent of their vulnerability varies greatly. Factors such as building architecture, design, orientation, materials, and ventilation can amplify indoor temperatures relative to outdoor temperatures during extreme heat events.^41^ With the increasing frequency and duration of heat waves, overheating and heat stress are the primary risks to buildings and their occupants as temperatures rise.^42^ High temperatures can induce strain and damage to the building system, for example, by reducing the lifespan of roofing materials.^43^ Heat affects the thermal expansion dimensions of materials and can lead to internal stresses that may result in cracking, bowing, buckling, and other forms of deformation.^44^, ^45^ Solar panels can overheat during periods of high temperatures and function less efficiently.^46^, ^47^ High temperatures could exceed the designed capacity of compressor‐based cooling systems. Heat waves can cause brownouts and blackouts,^48^ which in turn can lead to a loss of compressor‐based cooling.
- **Observed and projected impacts to building occupants**. Buildings can either moderate or exacerbate exposure to hazardous outdoor temperatures. They can also provide a place of refuge from elevated temperatures.^49^ The projected warming trend and greater frequency of extreme heat events may lead to higher utility bills for consumers because of the increased need for air conditioning.^50^ People with lower incomes may be less likely to have access to or use air conditioning. For example, while almost 90% of New York City residents have some form of air conditioning, fewer than half of public housing residents have access to it.^51^ According to a 2011 study in New York City, 34% of seniors or adults in poor health surveyed did not own or use air conditioning during hot days, 21% were unaware of heat warnings, and many did not consider themselves to be at risk of the extreme temperature exposures.^52^ Refer to Chapter 7, Human Health and Safety, for a detailed discussion on the health impacts of increased temperatures. Extreme heat events can cause hospital admission and death, particularly among older adults.^53^ People living in urban environments may be at a particularly increased risk from ambient heat exposure, as urban areas typically have higher heat indexes, or combinations of temperature and humidity, than surrounding suburban or rural areas.^54^ In the event of a power outage caused by extreme heat, residents can lose access to air conditioning, elevators, potable water, refrigeration, cooking appliances, and medical equipment.^55^ Without air conditioning, indoor temperatures can be much higher than outdoors, especially at night; this can continue for days after a heat wave.^56^ The New York City Department of Health and Mental Hygiene reported 96 heat‐stress deaths in the city from 2011 to 2020.^57^ Of those who died after suffering heat stress at home, 81% had no air conditioning and the rest had air conditioning that was either not working or not in use.^57^ Increased costs of indoor cooling systems put a disproportionate burden on low‐income households.^58^ The thermal envelope of a building moderates outdoor temperatures, while passive systems such as shading, insulation, and windows can protect occupants during a combined heat wave and power outage; this is known as passive survivability.^59^

#### 3.1.2 Sea level rise, coastal flooding, and seiches

New York State is extremely vulnerable to the effects of sea level rise, given that much of the state's coastline is highly developed and densely populated.^60^ The Long Island, New York City, and South Hudson assessment regions face this risk, as do portions of the Catskills and North Hudson regions that border tidally influenced portions of the Hudson River. Rising sea levels will increase the frequency and extent of storm surge and coastal flooding. Coastal areas of New York State are experiencing faster rates of sea level rise when compared with the global average—a trend that is expected to continue in the future.^31^ By midcentury, some coastal and estuarine locations in the state, such as the neighborhoods around Jamaica Bay, Queens, may experience monthly high‐tide flooding, also called “sunny day” or “nuisance” flooding, including flooded shorelines, streets, and basements.^31^ Sea level rise will also contribute to increased storm surge flooding and backup flooding in stormwater systems that drain to the ocean.^7^ Loss of nature‐based storm barriers will cause increased erosion that can affect buildings during storm events.^61^, ^62^

Communities bordering the Great Lakes face their own flooding risks due to fluctuations in lake levels and acute events called seiches. Seiches occur when strong wind and air pressure push water to one end of a large body of water. When the wind stops, the rebounding water causes flooding on the opposite end of the water body.^63^ In New York, seiches occur in Lake Erie and Lake Ontario and can damage shoreline structures.^9^ Seiche events occur every 1−2 years on Lake Erie and cause erosion on the lake's eastern shores.^64^ Some researchers have suggested that increased storm frequency and length will produce more and stronger seiches on the Great Lakes, while others have noted that reduced ice cover could lead to larger seiches and more associated damage during winter.^31^ However, there is an ongoing need to learn more about seiches and how they could change in a warmer future.^31^

- **Observed and projected impacts to buildings**. An increasing number of buildings and their systems will be vulnerable to coastal flooding as sea levels rise and the frequency and intensity of storms increase.^30^ Rising sea levels can cause temporary and permanent inundation of areas currently occupied by buildings.^7^, ^65^ Changes in the distance to the ocean, the water table, and increased salinity could cause the failure of foundations and other support systems, such as water and sewer systems.^66^ As sea levels rise, New York City's office, retail, and multifamily buildings could experience $582 million in estimated flooding damages (costs annualized for 2022).^67^ Coastal flooding (including flooding along the Great Lakes coasts) damages buildings through direct impacts to their structural integrity and water damage to electrical and mechanical systems.^68^, ^69^, ^70^ Floodwater velocity dramatically affects how flood levels of different depths affect buildings.^68^ Debris such as building materials, vehicles, and trees can be carried into buildings and destroy windows and other building components.^69^ Water and waves can soak wood materials and erode the soil around building foundations, thereby undermining foundations.^69^ Floodwater can float the building and building components.^69^ Water height and differences in pressure inside and outside the building can damage building walls.^69^ Erosion around the foundation of the building can compromise the foundation and affect the overall structural integrity of the building.^68^ Alternatively, floodwater can deposit sediment around and in the building, causing further damage.^69^, ^71^ Repeated flooding also shortens the lifespan of buildings. Chemical contaminants that might be present in floodwaters can make building surfaces toxic; corrode surfaces; and cause mechanical, electrical, and plumbing systems to malfunction.^69^, ^72^
- **Observed and projected impacts to building occupants**. Sea level rise and coastal flooding can also affect building occupants by affecting the availability and cost of flood insurance; costs for water management infrastructure; and the economic, social, and cultural costs associated with property damage and loss.^7^ In addition, moisture in indoor environments can lead to contaminants such as mold that can increase the prevalence of respiratory illnesses.^73^

#### 3.1.3 Changes in precipitation

As the climate warms, total annual precipitation in New York State is projected to increase, and so is the frequency of extreme precipitation (days with at least 1, 2, or 4 inches of rain).^31^ Increased precipitation—particularly heavy rainstorms—could result in a greater risk of flooding and storm damage.

##### 3.1.3.1 Inland flooding

Riverine or fluvial flooding occurs when water overtops streams and river channels and spills into adjacent land and buildings.^74^, ^75^ It can also result from dam failures and the release of ice jams.^7^ Urban or pluvial flooding occurs when rainfall and stormwater exceed the capacity of the stormwater/sewer system and back up into the city's built environment.^76^ In places with aging infrastructure, like many cities in New York, combined sewer systems (where sanitary sewers also capture and convey stormwater) create chronic and predictable flood areas.^76^ All regions of New York State experience flooding, but urban areas with a high percentage of impervious surfaces that limit underlying soils from absorbing water, as well as areas adjacent to valleys, are especially prone to flooding during heavy rainfall.^77^ Various studies have projected that extreme river flows and flood damages will increase in New York State during this century.^31^ The events of August 28, 2011, provide an example of the damage that heavy rain can cause, as heavy rainfall from the remnants of Hurricane Irene caused the Au Sable River to rise around 11 feet over its flood stage in the Adirondack towns of Keene, Keene Valley, Upper Jay, Jay, and Au Sable Forks. This flood caused substantial damage to residential, commercial, and community buildings, including the destruction of 24 homes and the flooding of Upper Jay's town library and the town of Keene's fire station.^78^, ^79^

Increased flooding and expanding floodplains, particularly along the coastal plain where flooding can be exacerbated by compound events such as the combined effect of rainfall and coastal flooding, will put more buildings and communities at risk in the future.^80^ Cities with aging infrastructure will be at a higher risk for stronger and more frequent flooding.^76^

- **Observed and projected impacts to buildings**. Damage to buildings because of inland flooding is similar to the damage caused by coastal flooding described above. However, the impacts of coastal flooding can be more severe due to the compounding effects of wave and tidal action, wind, and inland flooding. Building damage from inland flooding can be more severe when heavy rainfall results in compound flood events such as drainage system failure, levee failure, river overtopping, and flash flooding.^75^
- **Observed and projected impacts to building occupants**. Impacts to building occupants from inland flooding are also similar to the impacts caused by coastal flooding (refer to the discussion in Section 3.1.2).

##### 3.1.3.2 Winter precipitation

Snowstorms, ice events, and cold waves are prevalent across New York State, but the severity and frequency of events is projected to shift with a changing climate.^31^ Winter temperatures are expected to rise statewide throughout the 21st century.^31^ As a result, most of New York is projected to experience a reduction in snowfall, snow depth, and the length of the snow season.^31^ The exception is lake‐effect snow, which models project will increase in the next few decades as the Great Lakes warm and spend fewer days frozen, but then decrease later in this century as more of this precipitation falls as rain instead of snow.^31^

- **Observed and projected impacts to buildings**. While projected winter warming could reduce the likelihood of snow as precipitation increasingly falls as rain, increased building damage could occur from freeze‐thaw and rain‐on‐snow events as temperatures hover around the freezing point.^81^, ^82^ Older buildings, poorly maintained buildings, buildings with insufficient insulation, structurally deficient buildings, and buildings with flat roofs will all be at greater risk of damage.^81^
- **Observed and projected impacts to building occupants**. When buildings lose power as a result of severe winter weather, residents can lose access to potable water, heat, elevators, cooking appliances, and life‐sustaining equipment.^55^ Fires related to the use of space heaters or stoves for heat can kill and injure people, and poor ventilation of fuel‐burning heat sources increases respiratory illnesses, especially in children and people with asthma.^83^ Roofs that are not designed for large snow loads can release snow onto people and injure or kill them.^84^ Winter‐related maintenance is expensive and can strain household budgets.^85^, ^86^ Snow removal and winter maintenance can also strain municipal budgets.^87^

#### 3.1.4 Extreme events

##### 3.1.4.1 Cyclonic storms

Cyclonic storms include hurricanes, other tropical storms, and nor'easters. These storms produce high winds, heavy precipitation, storm surge, wave battering, and flooding, which can cause considerable damage to both coastal areas and areas far inland.^88^ Historical trends and climate model projections suggest that as the century progresses, New York State will experience increased tropical cyclone hazards such as high winds and coastal and inland flooding.^31^

In October 2012, Superstorm Sandy hit coastal New York and New Jersey, causing 10‐ to 11‐foot floods throughout large areas of Manhattan, Brooklyn, and Staten Island, killing 43 people and inundating more than 88,000 buildings.^7^, ^89^ In September 2021, heavy rainfall from Hurricane Ida fell in New York City at the rate of up to 4.1 inches per hour.^90^ The storm damaged approximately 33,500 buildings. Much of the damage was concentrated in lower‐income and immigrant communities of Queens, Brooklyn, and the Bronx with a high percentage of at‐risk populations. Most of the damage in small residential buildings (with one to four units) was from flooding in sub‐ or at‐grade space (e.g., basements, ground floors).^91^ In New York City, more than a dozen people died, most of whom were trapped in flooded basement apartments.^92^

Erosion, storm surge, flooding, high winds, heavy rain, and storm‐related debris cause severe damage to buildings and communities. Cyclonic storms compound the impacts of these hazards and severely compromise buildings and render them unsafe for occupation and use.^93^, ^94^ Superstorm Sandy offered many lessons related to the impacts of these storms on buildings,^95^ including damage to high‐rise buildings.^96^ Structures located in coastal zones, floodways, and designated flood areas are at higher risk of damage.^97^

##### 3.1.4.2 Convective storms

Convective storms and associated weather events include thunderstorms, tornadoes, hail, rain, strong wind, lightning,^98^ and derechos, which are long‐lived wind storms associated with bands of rapidly moving showers or thunderstorms in which damage typically occurs in one direction along a relatively straight path.

Although the effects of climate change on convective storms are uncertain, particularly around wind and lightning, the impacts of these storms on buildings can be significant and should be considered in resilience discussions. Tornadoes and high wind can damage and destroy manufactured (i.e., mobile) homes, particularly older models and those that are not properly anchored to the ground.^99^, ^100^ Wind enters through exterior doors and windows, and once inside, creates internal pressures that can damage the roof and walls and further expose the building and its occupants to damage and injury.^101^ Tornadoes, wind, and hail can also cause severe roof damage or failure.^102^, ^103^ Lightning strikes can cause substantial damage to buildings by igniting fires or overloading electrical systems.^104^, ^105^ The July 1995 Ontario/Adirondacks derecho contained wind gusts of 100 mph or greater at several points along a band from Jefferson and St. Lawrence Counties through the Adirondacks region and into Central and Eastern New York State, where vehicles and buildings sustained nearly $190 million in damage, including damage to mobile homes that were overturned.^106^

##### 3.1.4.3 Drought and wildfire

New York State is expected to receive more precipitation overall, but with shifting patterns that could lead to seasonal summer droughts becoming more common.^31^ Hotter and drier summer conditions, low fuel moisture content, and elevated “fire weather” could lead to an increase in the conditions that cause more frequent and larger wildfires—whether caused by humans or lightning strikes.^7^ Projections for the Northeast indicate that the fire season will begin earlier in the year, peak earlier, and last longer in the decades ahead.^31^ Wildfire occurrence is also projected to increase in New York State, but the absolute projected change is small, even under the highest greenhouse gas emissions scenario.^31^

Wildfires along the wildland‐urban interface threaten buildings, with the buildings providing a source of fuel.^7^ This problem could become more pronounced in areas where increased construction is occurring along the wildland‐urban interface. Rapid growth in these areas often results in more wildfire ignitions.^107^ Transmission lines can be the cause of wildfires when conditions are dry and winds are high. Conductors coming into contact with dry vegetation has been shown to be the major ignition cause of utility‐related wildfires.^108^ Embers and smoke from wildfires enter through mechanical systems, windows, vents, or doors.^109^ Fire can partially or completely destroy buildings and raise the risk of smoke inhalation, burns, injury, and even death for building occupants. Displacement due to fire causes extreme emotional and financial hardship to affected individuals and communities.

#### 3.1.5 Other climate‐induced impacts

##### 3.1.5.1 Indoor air quality

Indoor air quality refers to the characteristics of air contained in buildings, including humidity; temperature; and the presence of gases, particulate matter, and infectious agents.^110^ Issues with indoor air quality can result from a single source or a combination of many factors. Poor indoor air quality can originate from flaws in building design; failure of the building enclosure or envelope (e.g., roof, façade, foundation); or insufficient preventative maintenance of building envelopes, plumbing, and heating, ventilating, and air‐conditioning (HVAC) systems. Some problems are a consequence of the building's location and mixed uses of the building. Many typical indoor air quality issues are related to improperly operated and maintained HVAC systems; overcrowding; radon; moisture infiltration and dampness; and the presence of contaminants produced internally from sources such as cleaning supplies and aerosol products, mechanical equipment, and off‐gassing from materials in the building.^111^

Climate change influences levels and locations of outdoor air pollutants such as ground‐level ozone and particulate matter, as the Human Health and Safety chapter discusses in more detail. Air quality concerns associated with climate change include ozone formation, smoke from wildfires, wind‐blown dust, and increased use of pesticides (the latter is further discussed in the Agriculture chapter).^112^ Rising temperatures and carbon dioxide levels also promote plant growth, leading to the release of airborne allergens such as pollen. As pollutants and aeroallergens infiltrate homes and other buildings, indoor air quality will be adversely affected.^113^

Poor air quality can result in serious human health consequences through harmful cardiovascular and respiratory effects. On average, Americans spend nearly 90% of their time indoors,^114^ and changes to indoor air pollutant concentrations can, therefore, have serious health consequences. The adverse health effects of indoor air quality are well documented and can often be worse for human health than outdoor air quality.^115^ Concentrations of some indoor air pollutants are often two to five times higher than normal outdoor concentrations.^114^ Climate change will exacerbate the impacts of indoor air quality on health.^73^

Poor ventilation can lead to moisture, mold, and poor air quality.^116^ Humidity affects the formation and release of contaminants into indoor air.^117^ Expansion of growing seasons due to climate change can increase buildings’ vulnerability to rot.^118^ Indoor air quality also affects the aerosolization of building materials and compounds. Moisture can induce mold growth, which breaks down building materials.^119^, ^120^

##### 3.1.5.2 Pests

A hotter, wetter climate is conducive for rodent and insect pests to thrive.^121^ Warmer temperatures, increased precipitation, and extreme weather events will affect the distribution of pests and their impact on buildings and the environment.^121^, ^122^, ^123^ Projections note an increase in pest activity in New York State, making more buildings vulnerable.^3^

New York State's buildings are susceptible to insect or rodent pests.^88^ Extreme weather events often damage building envelopes,^88^ impairing their ability to keep pests out. For example, buildings will be at a greater risk for water damage, which can facilitate the infiltration of pests through small openings in the building envelope assembly.^73^, ^124^ Extreme weather also drives pests to seek shelter indoors, causing damage as they dig or chew their way in.^124^ Rodents are more likely to be found in buildings that offer access, shelter, and food.^125^ Rodents themselves can compromise building envelopes, systems, and foundation elements.^126^ Urban landscapes that have been abandoned after extreme weather events serve as homes and breeding grounds for rodents.^127^ Storm‐downed trees and debris can provide both shelter and food for rodent and insect pests.^127^ Insects that feed on wood can compromise structures and other building elements over time.^128^ Termites will have a greater impact on older, wood‐framed buildings.^3^ Structures that have previously been compromised by pests are likely to be further compromised.^124^

Residents of urban structures are at greater risk of pest‐related climate hazards. The density of the building stock in urban areas makes it difficult for pest control operators to manage services, and the demand for pest removal outweighs the available supply, further exacerbating pest problems. Additionally, urbanization compromises an ecosystem's ability to naturally control pests, thus increasing the demand for pest management.^129^

People in communities with environmental justice concerns are more likely to live and work in inadequate and/or older buildings and are, therefore, at greater risk of experiencing the negative impacts of pests, including health problems and housing insecurity.^124^ These populations are more likely to rent, leaving them with less control over pest control management in their living spaces.^130^ Racial segregation and urban disinvestment have resulted in many members of these communities being blamed for pest infestations as if they are the cause and not a symptom of a larger pest control problem.^131^, ^132^ Pests are not only influenced by the physical or ecological makeup of the city, but also by discriminatory land use regulations and housing policies.^131^ Older adults are also at higher risk of impacts from pest infestation because they are less able to relocate.^133^

### 3.2 Impacts to communities

Buildings are a form of social infrastructure that provide refuge from harsh weather conditions. Their capacity for resilience is critical to a community's ability to recover from severe weather events. In urban areas such as New York City, built infrastructure is the main variable that determines the extent to which climate change affects communities.^134^

Extreme weather events and disasters such as winter weather, storms, and flooding can damage and even destroy buildings and affect building occupants. Table 4-4 illustrates how these impacts accumulate at the community level.

**TABLE 4-4:** Community‐level impacts of climate change.

Category	Impacts
Economic and financial	Money spent on cleanup and repair of buildings becomes unavailable for other household needs. Local governments may need to cut other programs and services to pay for flood recovery. If businesses close permanently, it can lead to long‐term economic losses.^135^
Environmental and public health	Damaged buildings release contaminants into the air and water.^136^ Disasters disrupt critical infrastructure systems, including transportation, energy, telecommunications, public health, and wastewater treatment.^30^, ^137^ Extreme events disrupt daily living routines and can affect residents’ mental health.^138^
Social	Extreme events disrupt community political structures and social networks.^139^ Buildings with important cultural heritage value are vulnerable.^140^, ^141^, ^142^ There are disproportionate impacts to the rental housing market, low‐income renters, and households of color or that speak English as a second language.^143^ Those living in poverty may be less able to prepare for or respond to extreme weather events. Extreme events can result in a decrease in homeownership.^144^

### 3.3 Cascading and cross‐sector impacts

Buildings are part of a complex, interconnected web of energy, water, transportation, environmental, natural, and social systems.^145^ Impacts in other sectors can cascade to the buildings sector, and building‐related impacts can cascade to other sectors. For example, during Superstorm Sandy, loss of power to gas stations prevented occupants from being able to operate portable generators.^146^ In some homes, this affected human health because occupants could not operate their heating systems and were, therefore, exposed to low temperatures. This disruption to the electrical grid cascaded through the energy sector to gas stations and then to homes, eventually affecting human health.^147^

Buildings, building occupants, and communities are especially vulnerable when two or more acute events happen in succession—for example, when a power outage from a severe storm is followed by a heat wave.^148^ Nine days after Superstorm Sandy, an early winter storm delivered several inches of snow, temperatures in the 30s, and wind gusts over 50 miles per hour.^149^ Buildings in zones affected by Sandy were ill‐equipped to protect occupants from this subsequent round of winter weather. These kinds of compounding events could become more frequent in a changing climate.

Within buildings themselves, structural, mechanical, electrical, and plumbing systems interact to provide services to occupants. Because of the integration of these systems, a failure in one system can cause a cascading impact that leads to the failure of other systems within the building. For example, New York City's water supply, which relies on gravity‐fed aqueducts from the Catskill‐Delaware and Croton reservoirs, provides sufficient water pressure for only the first five stories of occupancy.^150^ Electric pumps are needed to pump water above the lower floors. During power outages caused by Superstorm Sandy, buildings over five stories in affected areas could not provide water or sanitary services to the upper floors.^151^ A damaged school can also indirectly affect a community by reducing economic output when parents need to be absent from their jobs to care for their children when schools are unoccupiable.

### 3.4 Regional differences

Every county across the state is at risk from climate‐driven hazards already, and a long‐term examination reveals increased vulnerability over the next century. No location or building type is immune to the impacts of climate change. Nonetheless, New York does have regional differences that influence the impacts of climate change on buildings, including the likelihood of hazards, building types, industry, and construction capacity.

#### 3.4.1 Geography of hazards

The geography of the hazards described in Section 3.1 varies by region. For example, sea level rise mainly affects the New York City, Long Island, and South Hudson assessment regions, along with portions of a few other regions along the tidally influenced stretch of the Hudson River. Coastal flooding affects those regions in addition to the Great Lakes region. Cyclonic storms are more likely to affect regions closer to the ocean, though other areas are also at risk for secondary weather from these events. The density of population in the New York City metropolitan area makes residents especially vulnerable to large hurricanes.^152^ Changes in temperature, heat waves, precipitation, indoor air quality, convective storms, inland flooding, and pests will affect all regions. Changes in winter weather will be more likely to affect the state's colder, snowier regions, including the Great Lakes, the Champlain and St. Lawrence valleys, and the Adirondacks.

#### 3.4.2 Building types and industries

Building types and industries vary by region, which affects how and to what extent buildings are damaged by climate‐related events. For example, Superstorm Sandy disproportionately damaged or destroyed low‐rise stick frame houses, while high‐rises lost function of basement‐level building systems.^146^ High wind and tornado events injure and kill disproportionate numbers of residents of manufactured homes nationwide.^153^ Hail damage or destruction of agricultural facilities can threaten agricultural buildings, livestock, and crops.^154^ Spills of hazardous materials stored on farms can contaminate the environment and animals. Farms are more prevalent in the Great Lakes and Central/Finger Lakes assessment regions.

Table 4-1 in Section 2.1 summarizes building stock by assessment region, including information about each region's building count, building area, and value. Figure 4-2 in the same section maps the percentage of the state's buildings by region.

#### 3.4.3 Adaptive capacity and construction capacity

In the buildings sector, the most prominent economic impacts from future climate impacts, aside from the direct impacts on the businesses occupying buildings, will likely affect the real estate, insurance, and construction industries.^155^, ^156^, ^157^, ^158^ While it is hard to predict the impacts on highly volatile, market‐based industries like real estate and insurance, it is possible to estimate the construction industry's ability to prepare buildings for future weather events or rebuild damaged buildings after an event.

Construction capacity varies from region to region. Regions with more capacity, often around metropolitan areas, tend to be better able to repair buildings after extreme events, make adaptive modifications to existing buildings, or construct new buildings designed to resist future climate hazards. For this assessment, regional construction capacity is defined as the supply of construction professionals, including employees and firms, within each region.^3^ Construction capacity is measured by the number of square feet of building space that each construction professional or firm would theoretically be responsible for in a mass recovery campaign that affects all the buildings in a region.^3^ While such an event is unlikely and outside help would likely be needed, it provides a means of comparing capacity across all regions in the state. Regions with a lower count of building square feet per construction firm have a higher construction capacity because there is a greater ability to quickly mobilize local labor and rebuild after an event.

Preparing the buildings sector for future climate hazards can have benefits beyond simply lowering the sensitivity of buildings to extreme hazards. Sustained preemptive actions can also build local capacity for recovery activities after weather events, including events that occur under the current climate. Increased local capacity to recover after an event would reduce overall recovery times and minimize economic losses in each region.

Sources of funding are important as well. Hazard mitigation projects funded by outside sources have the additional advantage of creating new economic output while they build local capacity. This may be particularly crucial for less wealthy regions where local governments and property owners may not have the funding or capacity to handle such projects. Some regional economies may be better at generating spin‐off returns from construction spending than others. For example, the Southern Tier and Adirondacks regions rank lowest in the state in terms of their ability to generate additional returns on construction spending.^3^ However, all regions will see some new spin‐off returns on projects funded by external sources.

## 4 VULNERABLE POPULATIONS AND SYSTEMS

### 4.1 Communities at risk

A community can comprise connected or organized groups of people who share a common geography and sense of place, or share common interests, perspectives, characteristics, interests, and/or goals.^159^ Because geography and race are intersectional, the terms “population” and “community” are used interchangeably in this section.

People of color, Indigenous Peoples, and low‐income individuals and families are more likely to live in communities with buildings that have greater exposure to climate hazards. The effects of climate change will disproportionately burden these socially vulnerable populations—often referred to as frontline communities—that experience the “first and worst” consequences of climate change.^160^ Frontline communities are disproportionately exposed to climate risks not only because of historic, present, and future environmental risks, but also because they possess fewer economic, social, and/or political resources to respond to those risks or the root causes of socioeconomic marginalization.^160^

Vulnerable subpopulations within communities at risk from the impacts of climate change include adults over the age of 65, children, and the chronically ill. Specific impacts of climate change on the health of older adults and children are discussed in the Human Health and Safety chapter. It is important to note that certain building types, such as nursing homes and K–12 schools, have high numbers of these vulnerable populations; these buildings are discussed in greater detail in Section 4.3.

Vulnerable communities and associated structures include those located in river valleys, coastal zones, or other areas identified as floodplains.^94^, ^161^, ^162^ Renters and people living in subsidized housing are more vulnerable to displacement because they lack the financial resources to readily relocate.^163^

### 4.2 Community systems at risk

Risks to buildings not only threaten individual lives, but also pose threats to community‐level resilience because buildings intersect with community functions. The systems of a community that involve buildings are illustrated by the Community Capitals Framework, a schema that suggests that the “lifeblood of any community can be linked to the presence and strength of seven community capitals, resources that can be invested or tapped for the purpose of promoting the long‐term well‐being of communities.”^164^, ^165^ The seven types of community capital are as follows:

- **Built**. Buildings are usually connected to all infrastructural systems of a community, especially water and sewer systems, energy utilities, broadband and other information technologies, and transportation networks. Vulnerably located community infrastructure systems can experience climate‐related impacts like increased water exposure from sea level rise and corrosion from salinity, which can lead to the destruction of critical communication lines, thereby compromising access to care or shelter in emergencies.^166^, ^167^ Furthermore, buildings are integral components of all forms of critical infrastructure. While these interconnections might demonstrate vulnerability when damages occur in any one sector, they also offer opportunities for adaptation to be multisectoral.
- **Natural**. Nature creates the sources of energy and materials used for building; in turn, humans’ design decisions can dictate building performance and impacts on natural systems. For example, to reduce the effects of sea level rise, many homeowners and communities construct sea walls and other physical structures, but these structures can also impede natural ecological processes.^168^ Climate change will produce more intense storms, which will lead to more corrosion, waste, and debris. As buildings decay or are torn down, especially when materials are disposed of improperly, they can emit toxins and pollute soil, vegetation, and water, affecting the quality of the natural environment.^169^, ^170^ As the Ecosystems chapter explores in more detail, climate change will also affect local production of wood‐based building materials as harvests are affected by invasive species, weather, and ground conditions for logging.^171^
- **Cultural**. Buildings are produced at a specific time and place and, therefore, carry a unique historical or cultural significance. Many unique architectural details cannot be recaptured or retained after sections of a building are damaged. Climate threats that will alter buildings include warping from heat exposure; foundational damage due to flooding, sea level rise, and/or land subsidence; and wind damage from storms.^172^ Increasingly, scholars are reporting on the impacts of climatic stressors on historical buildings, archeological sites, monuments, and other cultural assets. One study^173^ cataloged the impacts of climate change on cultural heritage, summarized in Figure 4-6. For instance, changes in moisture levels in historic building materials can lead to increased decay and efflorescence, which can result in cracks and color changes on historic façades. The ways in which properties will accommodate climate resilience measures to buildings vary by community. Some structures will be elevated, and others will not, while some lots might become vacant. The character of the community will change, and these changes, especially when compounded with demolitions such as those from flood buyouts, can lead to a highly altered landscape with a corresponding loss of heritage and social belonging.^174^
- **Human**. Climate effects such as extreme heat, drought, and flooding affect building structures, and these effects can influence human health and wellbeing. For instance, increased precipitation can foster mold growth that can worsen occupants’ asthma and allergy symptoms, and living in homes without air conditioning can cause residents to experience dehydration and heat‐related illness. A degraded home environment or the economic stressors from costly repairs could also affect residents’ mental health and lead to psychological stress or imbalance.^175^ Repairs also divert people's time and money away from other productive pursuits.^164^
- **Political**. As residents leave hazard‐prone regions, their movement has the potential to change the political landscape of both the sending and the receiving communities.^176^, ^177^ A loss of residents might trigger municipal disinvestment through service reduction, and high rates of vacancy and fewer services might depreciate home values, thereby leading to a further decline in market value for remaining residents.^178^, ^179^ Receiving communities may be able to develop opportunities because of the new population growth, or, conversely, they may need to mediate potential conflicts.
- **Financial**. Buildings are substantial investments, and the costs of adaptation require considerable financial equity. Homes are many Americans’ most substantial assets and mechanisms for building intergenerational wealth,^180^, ^181^ so climate risks to homes threaten the future financial security of individuals and families. Because the costs of both displacement and reconstruction are substantial, renovations and resilience measures taken as soon as possible will be more affordable than those performed later, especially after storms.
- **Social**. The threats of climate change to buildings can profoundly affect social networks and potentially reduce the social capital needed for community resilience. As structures become degraded, they become less desirable or even nonfunctional spaces for social activities. The reduction or cessation of social activities could reduce bonds among community members. When buildings must be vacated, the relocation of residents could lead to the erosion of vital community‐based social networks.^182^, ^183^ Youth might lose touch with their cultural heritage and sense of place.

**FIGURE 4-6:**
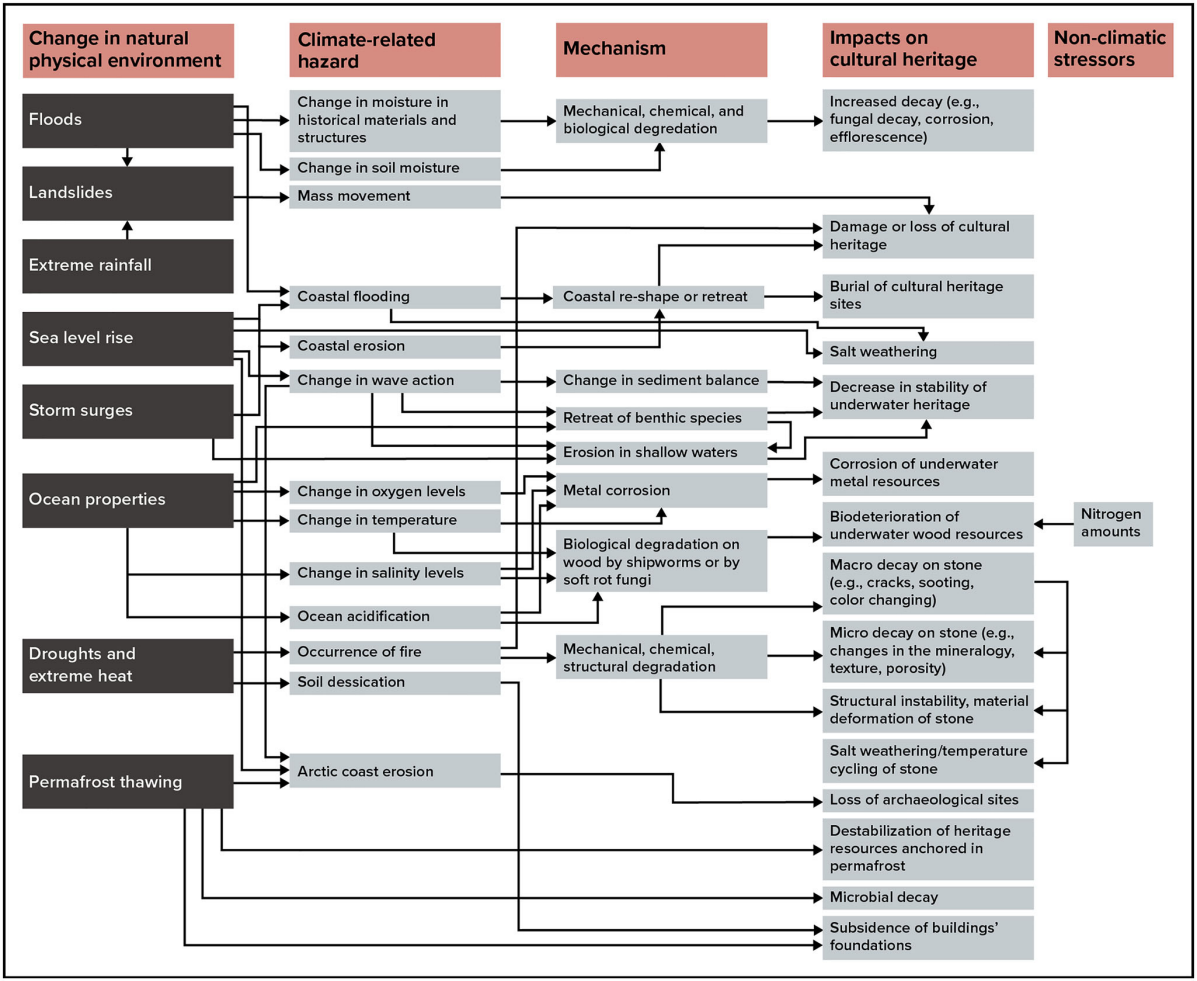
Changes in the natural physical environment affecting cultural heritage. Adapted from Sesana et al.,^173^ licensed under CC BY 4.0.

#### 4.2.1 Resources to support a just transition

As the impacts of climate change occur across New York State, it is important that residents of frontline communities have access to a built environment that can adapt to the expected changes in the climate and is resilient to extreme weather events. As outlined in Section 5, the technologies needed for adaptive and resilient buildings already exist. However, many frontline communities do not have access to capital to implement these strategies for their buildings and communities. Without policies and targeted financial resources, frontline communities will continue to experience climate injustice, as discussed in Section 2.4.

A pathway to improve the quality and standard of buildings in frontline communities is to build affordable new buildings for residents of these communities.^184^ Another pathway is to retrofit existing buildings. Many residents of frontline communities occupy existing buildings that are substandard and at risk of exposure to the impacts of climate change. These residents are either building owners without access to capital for critical retrofits or renters who are not authorized to make improvements to the building. For owners, resources and programs are available to help support building retrofits, but often these programs do not meet all the needs of residents in frontline communities.

### 4.3 Building types at risk

Based on the latest climate projections for New York State, all building types will be subject to increased intensity of storms, more days with extreme temperatures, and rising sea levels and/or increased flooding in coastal zones and floodplains. Strategies related to improving building ventilation, roofs, roof drainage, foundations, and building operations and reducing the urban heat island effect apply to all building types. Buildings that provide education, housing, health care services, and manufacturing must be designed, built, and operated to provide a high quality of life for occupants; support occupant and community health and wellbeing; and add value to the community's health, environment, and economy.

Each building type discussed in this section has its own vulnerabilities to climate impacts and occupants with special needs. Some buildings in high‐risk categories or that serve critical functions after a climate‐related event will need a more resilient design to shield them from cascading risks. For example, a hospital will need to ensure occupants remain safe even when the power is out (passive survivability). Some building types serve as emergency response centers and emergency shelters for house‐fragile populations, such as children in schools or older adults in nursing homes.

Specific building types and their particular vulnerabilities to climate change impacts are described below.

#### 4.3.1 K–12 schools

Schools are critical buildings within communities.^185^ Students spend one‐third of their waking hours learning, playing, and growing in schools, and quality environments are needed to maintain children's health, student enrollment, and educational outcomes.^186^ Climate impacts to schools include increased levels of extreme heat that threaten indoor air quality and bring discomfort that can affect student learning and staff working conditions.^186^ Closures due to climate‐related winter storms, flooding, and other climate hazards affect students’ learning and physical and mental health.^187^, ^188^ Moreover, school buildings and campuses often provide key community functions, including serving as emergency shelters and emergency response headquarters. In extreme weather events, schools can act as shelters for displaced or vulnerable residents. They can also operate as distribution centers for food, essential services, and supplies for communities in crisis.

A combination of strategies such as building performance modeling, insulation, wind protection, passive building systems, and redundant building systems could be considered to make school buildings adaptive to climate change and resilient to climate hazards.^186^ In addition, comprehensive indoor air quality and enhanced fire protection strategies can help school districts prepare their buildings for climate change impacts.^186^ These strategies would be beneficial for severe storms, winter weather, heat waves, floods, sea level rise, and wildfires.

#### 4.3.2 Medical facilities

Medical buildings are a critical part of the emergency response to various outcomes of climate‐related impacts. For instance, when Superstorm Sandy struck the New York City region in 2012, Bellevue Hospital had to temporarily move patients elsewhere.^189^ A combination of gray/green infrastructure, flood protection, wind protection, redundant building systems, backup power, emergency management, and passive and active building systems could help medical buildings not only adapt for severe storm winter weather, heat waves, hurricanes, floods, and sea level rise, but also reduce greenhouse gas emissions.^190^ As airborne diseases such as COVID‐19 become more prevalent, medical facilities must also prioritize enhanced indoor air quality strategies.^191^

#### 4.3.3 Manufactured homes

There are 192,890 manufactured housing units in New York State, representing 2.4% of the state's total housing stock; however, in rural areas, that percentage jumps to 10.3% of housing stock—more than 100,000 manufactured housing units (Borges M, New York State Rural Housing Coalition [2023, May, Personal communication]). Section 3.1 notes that manufactured homes are more vulnerable to windstorms because of how they are constructed and anchored to the ground. Moreover, manufactured homes are often disproportionally located in floodplains or other areas with relatively high exposure to natural hazards, as negative perceptions of manufactured homes and communities can influence planning and zoning practices that relegate manufactured home parks to marginalized parcels within communities.^192^

Manufactured homes on high‐ and moderate‐risk lands face substantial barriers to financing adaptation efforts. Repair costs can far exceed manufactured homes’ values, particularly for older manufactured homes; thus, disaster recovery aid can often prove insufficient for repairing or replacing the structures. While residents of manufactured home parks typically own their individual homes but rent the land underneath,^100^ relocation is difficult. Moving costs are sizable, and few vacant lands are available, as municipalities have zoning practices that restrict manufactured homes, and corporate landlords and investors are purchasing many manufactured home parks.^193^

#### 4.3.4 Nursing homes and institutional dormitories

Nursing homes (also known as long‐term care facilities) and institutional dormitories (which include emergency housing and correctional facilities) are critical because of the vulnerable nature of their occupants. Approximately 117,000 people currently reside in the state's 610 nursing homes.^194^ An aging population coupled with predicted longer life expectancies will likely increase the demand for nursing home facilities. By 2035, approximately one‐fifth of New York's population is expected to be 65 and older, compared with 16.4% in 2019.^195^ Because of age and underlying health conditions, nursing home residents are particularly vulnerable to climate hazards such as flooding and extreme heat and cold.^196^, ^197^, ^198^ Nursing home facilities’ ability to withstand the impacts of climate change can mean life or death for residents. A loss of power or flooding caused by climate exposes residents to climate hazards, worsening underlying health conditions and often requiring evacuation or rescue of residents.^196^, ^197^, ^198^ Well‐built, modern facilities can often withstand power outages and flooding events and have staff and contingency plans to handle them. However, older facilities, often serving low‐income residents, may not have the capacity to cope with a loss of power or flooding caused by climate events.^197^

Climate change presents many challenges for New York State's correctional facilities. Extreme heat is especially dangerous for incarcerated populations. For example, some correctional facilities in the state have air conditioning only in select areas (e.g., medical units) but not in other areas such as housing cells.^199^, ^200^ Most prisons have limited facilities for medical emergencies,^201^ and access to external urgent care might be impaired under severe flooding conditions. Additionally, correctional facilities with deteriorating infrastructure pose safety concerns for incarcerated populations due to the presence of environmental health hazards, such as asbestos and mold.^202^ It is likely that flooding and storm events will contribute to further degradation of correctional buildings and, in turn, to health and safety risks for incarcerated populations. There is also a demonstrated need for departments of corrections to update or create evacuation plans for prisoners during storm events.^203^

Emergency management, flood protection, wind protection, green/gray infrastructure, and redundant building system strategies could be beneficial ways to adapt nursing homes and institutional dormitories to severe storms, winter weather, heat waves, hurricanes, floods, and sea level rise. Comprehensive indoor air quality strategies, integrated pest management strategies, and enhanced fire protection strategies could also help to protect these buildings and their vulnerable residents.

#### 4.3.5 Government facilities

Government buildings are essential to running the daily affairs of New York State and its municipalities, as well as responding to emergencies. General services buildings include offices and libraries, while emergency response buildings include firehouses, police precincts, and sanitation. For general government buildings, implementation of gray/green infrastructure, building performance modeling, potable water, fire protection, and flood protection would help mitigate severe winter weather, heat waves, hurricanes, floods, sea level rise, and wildfires. In addition to the recommended strategies for general services buildings, emergency response buildings can also benefit from redundant building systems, emergency management, potable water access, neighborhood development, and wind protection strategies.

#### 4.3.6 Agricultural buildings

Agricultural buildings such as barns, livestock pavilions, and greenhouses represent less than 1% of New York State's building stock (Table 4-2). However, these buildings are critical to the state's food supply and rural economies. New York is a leading producer of dairy products, maple syrup, wine, and other crops and products, so storm events like floods and droughts that disrupt agricultural facilities would also affect national supply chains. Refer to the Agriculture chapter for a more complete discussion of economic and social losses in farming and food production due to climate change.

#### 4.3.7 Religious buildings

Similar to schools, religious buildings are critical to communities and are often centrally located. Like schools, they can provide emergency shelter and operate as distribution centers for essential services or food. If a religious building is designated as an emergency response center or shelter for any community, a combination of strategies such as emergency management, water reclamation, and flood protection could be beneficial.

## 5 ADAPTATION AND RESILIENCE

How people design, construct, and operate buildings directly shapes society's capacity to manage resilience. It is neither technologically nor financially feasible to eliminate every climate threat that a building will experience. Strategies should be selected with consideration for a particular project's location, use, and construction to optimize risk protection with limited resources.^204^ Building types and their particular vulnerabilities to climate change are described in Section 4.3.

Greenhouse gas emission reductions, adaptation, and resilience in buildings are inherently intertwined. In the best‐case scenario, solutions address all three areas. For example, well‐designed, energy‐efficient buildings have lower associated emissions, provide greater comfort and indoor air quality, and increase passive survivability.^205^ In contrast, emission reductions without adaptation may be maladaptive. For example, a newly constructed building designed with a fixed‐glass curtain wall enclosure and a super‐high‐performing HVAC system may exceed code requirements and accrue good energy scores on green building rating systems, yet may still overheat during a summer power outage, creating a risk to occupants. One adjustment would be to include operable windows or shades. Conversely, air conditioning is frequently offered as a critical adaptation strategy to prevent heat‐related morbidity and mortality, but it increases energy use and greenhouse gas emissions. There are many passive cooling strategies (i.e., building cooling techniques in which no power is consumed) that can be folded into the siting and design of buildings to support occupants in a changing climate while reducing the burden on energy sources.^206^

Building designers and operators must consider the climate change impacts discussed in Section 3, including sustained stresses, such as increased average temperatures, and shocks, such as extreme weather events. Buildings and occupants are also vulnerable to system‐level failures, including damage to the electric power grid and water and sewer infrastructure. Designers and operators should consider buildings as part of a larger network, rather than solving problems by taking individual measures.

Buildings are the purview of a number of disciplines, including urban planning, architecture, and engineering, each of which frames the concepts of adaptation and resilience in different ways.^207^ Four main academic domains underpin the consideration of adaptation and resilience in the architecture, engineering, and construction communities: ecology, engineering, disaster risk reduction, and the social sciences.^208^ The resulting different definitions, methodologies, and heuristics can make interdisciplinary and cross‐sectoral adaptation efforts challenging because issues are framed in disparate ways or have solutions focused on one sector.

The following sections summarize key considerations for adaptation and resilience in the buildings sector that span all phases of the building process.

### 5.1 Building design and construction

#### 5.1.1 Passive design

Passive building design is a holistic approach that supports building energy performance with heating and cooling strategies that do not consume power. These approaches can greatly affect building energy needs throughout the lifetime of the building.^209^ Passive building systems can help reduce heat gain, interior air temperature, peak demand, annual electrical energy use, and annual energy cost.^210^ Passive building design strategies could include optimizing site potential through ideal building orientation, considering factors such as prevailing wind patterns and daylight availability. Sun shading devices and properly sized south‐facing windows reduce building heat gain and cooling requirements by reducing direct solar heat absorption from the sun in the summer,^211^, ^212^ while allowing heat gain in the winter months when the sun is lower in the sky. Daylighting can be improved by adding exterior windows and reducing the number of interior walls that block the flow of light.^213^ Natural ventilation relies on cross‐ventilation and the chimney effect to cool buildings.^214^

While passive design usually focuses on reducing building energy use, these strategies often have climate resilience cobenefits. Passive building systems can enhance passive survivability, which is defined as a building's ability to maintain thermal comfort during power outages.^205^, ^215^ As the frequency, length, and intensity of heat waves increase with climate change, passive survivability becomes even more important, particularly for vulnerable populations. Extreme heat events also have the potential to increase power outages, as spikes in summer peak electricity demand (caused by increased air conditioning use) strain the grid's ability to generate and transmit enough power to meet demand.^216^ By reducing cooling load on the grid, passive design strategies can help reduce the risk of power outages.

Passive building design can also help reduce climate‐related risks at a community scale. For example, green roofs can lower urban heat island effects, reduce cooling energy demand, and capture rainwater to reduce urban flooding.^217^

Passive design strategies for building envelopes include the following:

- **Insulation and airtightness**. Proper insulation and air sealing increase the efficiency of the building envelope by slowing heat transfer through exterior walls and roofs. These measures can help maintain comfortable interior temperatures while reducing the use of active building systems.^218^ During heat waves or in the case of a grid outage, insulation can help maintain safe indoor temperatures without active cooling.
- **Roofs**. Roofs absorb heat, which then radiates into buildings, increasing interior temperatures and reliance on active cooling systems. Roofs and other nonreflective surfaces also radiate heat back into the environment and contribute to the urban heat island effect.^219^ Roof coverings can control the amount of heat absorbed from the sun, reduce urban heat island effects, and reduce the need for active cooling systems in all building types.^220^ Cool roofs with high‐albedo coverings reflect energy from the sun rather than absorb it, while green roofs reduce the amount of heat absorbed and help reduce stormwater runoff.^221^
- **Windows**. High‐performance, energy‐efficient windows can reduce indoor air temperatures by reducing heat transfer through the window elements and by reflecting solar radiation.^222^ Integrating daylighting systems into building design reduces dependence on electrical systems that can fail during climate hazard events.^222^ The Urban Green Council's Building Resiliency Task Force recommends operable windows in all residential buildings so that natural ventilation can provide cooling when active building systems are unable to do so.^223^ Windows must also be designed and properly installed to prevent moisture and water from entering the building during and after severe storms, which can cause mold growth and lead to poor indoor air quality.

#### 5.1.2 Temporal prioritization

Whether and when to adapt to climate change is a complex decision that must consider a building's lifespan, functions, importance, and constituent materials and systems. As climate change risks increase throughout this century, every adaptation, retrofit, and new construction design decision must be based on climate projections and expected hazards. These design decisions must align with a building's or its systems’ life cycle to be cost‐effective, sustainable, and future‐ready.

New York State's new and existing buildings are expected to support many generations of use, while individual components of a building may be replaced more frequently. Adaptation is needed only for buildings or systems that will still be usable when the climate change impacts occur^224^; therefore, it is increasingly important to determine the feasible useful life of a building or system.^38^ Decision‐making about design interventions should account for the expected useful life of the component parts of a building to account for the appropriate level of climate risk.^225^ For example, the foundation or envelope elements of a residential building could last for many decades, while its HVAC system might last only 15 years. During the lifetime of the building, replacing equipment at the end of its useful life provides a relatively simple opportunity for adaptation, whereas making changes to the envelope could be much more complicated and thus even more important to prioritize during construction. New York City's Climate Resiliency Design Guidelines provide a good example of how to analyze climate risk for various building types using time and anticipated change as key factors in decision‐making and design interventions.^225^

Electrification of building systems will play a major role in greenhouse gas reductions as the electric power grid continues to add more renewable energy sources. As New York State considers pathways to enhance building resilience and adaptation, electrifying buildings also becomes increasingly important from an adaptation and resilience perspective. Electrification enables the integration of additional measures, such as integrated photovoltaics, electric vehicle charging infrastructure, battery energy‐storage systems, and demand response (DR) and grid interactivity. Implementing such measures will help meet New York State's energy needs and bolster the resilience of the state's energy systems.

### 5.2 Building operation and maintenance

With changing temperature and precipitation patterns, buildings will need to operate in conditions that exceed their initial design capacity. Proper and efficient facility operations and maintenance can help ensure that buildings remain operational during and after climate hazard events and reduce reliance on energy grid systems that can fail during climate hazard events.^226^ A comprehensive facility operations and maintenance manual and a life‐cycle cost analysis can identify sustainable and resilient practices that reduce cost and dependency in building systems.^227^, ^228^ Optimized operations and maintenance practices are also a critical resilience component.^229^ Emergency operation plans that incorporate evacuation into building operations allow more buildings to be used as shelters, relocation centers, and transportation support areas, and more effectively use community assets to coordinate responses.^230^

The U.S. Department of Energy defines a grid‐interactive building as “an energy‐efficient building that uses smart end‐use equipment and/or other on‐site DERs [distributed energy resources] to provide demand flexibility while co‐optimizing for energy cost, grid services, and occupant needs and preferences, in a continuous and integrated way.”^231^ Grid‐interactive buildings use load shifting strategies to support both long‐term grid planning/operation and emergency grid management. According to the *2025 California Demand Response Potential Study*,^232^ load shifting strategies fall into four main categories, which are coordinated between utilities and building operators:

- **Shape** captures DR that reshapes customer load profiles through price response or on behavioral campaigns—“load‐modifying DR”—with advance notice of months to days.
- **Shift** represents DR that encourages the movement of energy consumption from times of high demand to times of day when there is a surplus of renewable generation. Shift could smooth net load ramps associated with daily patterns of solar energy generation.
- **Shed** describes loads that can be curtailed to provide peak capacity and support the system in emergency or contingency events—at the state level, in local areas of high load, and on the distribution system—with a range in dispatch advance notice times.
- **Shimmy** involves using loads to dynamically adjust demand on the system to alleviate short‐run ramps and disturbances at timescales ranging from seconds up to an hour.

As described in the study, “shift” resources are often used to move flexible loads from morning and evening hours to the middle of the day when solar production is high. An example would be scheduling electric vehicle chargers, clothes washers and dryers, and dishwashers to run during off‐peak hours. In contrast, “shed” resources may be used during extreme weather events as the increased demand for air conditioning during heat waves can stress grid operations, leading to rolling brownouts or extended outages.^232^ An example of a shed event would be to ask customers to lower their demand for a brief window of time to reduce system stress during an extreme heat event.

### 5.3 During and after extreme weather events

Owners and operators of buildings that serve critical functions, such as hospitals, emergency shelters, and nursing homes, have a responsibility to ensure their buildings are safe, functional, and habitable for occupants during climate hazard events. Protecting building systems from extreme weather events is essential for ensuring that buildings will remain operational and continue to serve as centers of refuge (e.g., cooling centers). These systems, such as heating and cooling and wastewater processing systems, are expensive to repair or replace, so resilient building systems can save money in the long term while keeping buildings operational during and after events such as major floods.^233^

- **Emergency management**. Emergency management can help prepare communities for the impacts of climate hazard events, reduce potential damage, and support rapid recovery.^234^ Planners should understand that buildings serving critical functions play important roles in responding to emergencies and have the potential to protect vulnerable populations. Cohesive emergency management plans reflect and respond to the specific levels of sensitivity and exposure in their area, while ensuring their plan aligns with current state‐level response protocols.^235^ Proper emergency management for all populations requires up‐to‐date information, redundant building systems, and organization. These tactics are especially useful in dense urban areas when high concentrations of the population may be exposed to a hazard.
- **Flood protection**. Extreme precipitation events and sea level rise can overflow drainage systems and waterways with stormwater runoff, which can leave neighborhoods without access to emergency services and other critical services.^236^ Neighborhood flood protection helps communities prepare for the collective impacts of flooding hazard events and begins with understanding the community's current flood vulnerability. Using projected flood maps that better represent future flooding conditions can help communities in coastal areas, along the Hudson River, and in other flood‐prone areas throughout the state to better plan and prepare for future floods.^237^, ^238^ FEMA provides guides to help business owners and homeowners identify flood preparedness actions, such as disaster procedures, emergency communications plans, and insurance policies.^239^ Aging infrastructure systems are overburdened by sea level rise, heavy downpours, extreme heat, and other events that they were not designed to handle.^172^ As the Water Resources chapter explains in more detail, investments in both green and gray infrastructure can save money in the long term and reduce damage from future climate events. Gray infrastructure includes critical systems that provide sanitation and stormwater control for communities. With green infrastructure, stormwater is typically channeled into in a basin or ditch designed to allow the water to seep or infiltrate the ground and recharge groundwater supplies, or to slow its passage into storm drains during peak flow periods to avoid overwhelming the stormwater system.^240^ Green infrastructure supports a variety of climate resilience goals, such as replenishing groundwater reserves through ground infiltration and reducing the impacts of sea level rise^241^ by creating living shorelines.^242^, ^243^ It also reduces urban heat island effects, building energy demands, and energy spent managing water.^241^ Such systems can be implemented at the city and regional scale (e.g., wildlife habitat, wetlands, and infrastructure), as well as on individual properties (e.g., rain barrels and rain gardens, planting larger trees for shade and heat island mitigation, separating on‐site stormwater and sanitary systems).^244^, ^245^
- **Redundant building systems and backup power**. Redundant building systems ensure that critical building functions, such as electrical power, wastewater processing, and heating and cooling, remain available should a system be compromised. While redundancy may reduce overall efficiency, it will increase resilience and help prevent the negative impacts of system failures. Redundant building systems reduce the likelihood of a building shutdown and provide the opportunity to repair one system while the other is being maintained.^246^, ^247^ Providing alternative energy sources will help critical facilities remain operational during climate hazard events.^248^ These systems can provide code‐required emergency power (e.g., fire alarm, emergency lighting, and evacuation elevator use), as well as longer‐term power for limited elevator use (important for accessibility, seniors, and families with young children), domestic water pumps, access control, limited electrical plug loads, Wi‐Fi, and electric vehicle charging. To shelter in place, building occupants must have access to potable water. However, the water pressure from the street can supply water only to lower stories without supplemental pumping. This makes high‐rise buildings vulnerable to prolonged power outages.^249^, ^250^ Buildings can increase resilience by using backup generators or photovoltaic integrated hot water systems to maintain access to potable water. Energy can also be stored thermally (e.g., domestic hot water tanks), which can still be accessed if power failures occur.^251^

## 6 LOOKING AHEAD

The discussions throughout this chapter underscore the importance of buildings as more than just physical infrastructure. Buildings provide social infrastructure where people live, work, learn, help one another, and build community. This interconnectedness raises the stakes of climate change, as direct impacts to buildings can cause serious cascading disruptions. Yet, it also means that making buildings more resilient to climate change can yield many additional benefits for society. Resilient building design can also promote environmental and social justice and equitable access to services like housing and jobs, generating environmental, social, economic, and human cobenefits for occupants, neighborhoods, surrounding cities, and the larger environment.

Adaptation should not be approached as a one‐time event, but rather as a continuous process. An ongoing interdisciplinary process of updating climate hazards and identifying emerging technologies is needed to ensure that buildings are responsive and adaptive to current and future conditions. Addressing the impacts of climate change to New York's building stock will require the integration of diverse perspectives from engineers, scientists, designers, community members, and Indigenous leaders. Multidisciplinary solutions must ensure that all potential consequences are considered to mitigate unintended impacts to communities and those who are most vulnerable.

This section summarizes several opportunities for positive change: solutions that not only help to create more resilient buildings, but also simultaneously address other challenges and improve New Yorkers’ lives in additional ways. It then identifies emerging topics and research needs that could further improve understanding of buildings sector impacts and adaptation options in the future. The section concludes with a review of the major findings presented in this chapter.

### 6.1 Opportunities for positive change

Many of the adaptation strategies discussed in Section 5 can simultaneously advance both sustainability and resilience. For example, the architecture, engineering, and construction communities are beginning to adopt the concept of regenerative design, which embraces practices that reduce greenhouse gas emissions and help buildings adapt to a changing climate.^252^, ^253^ Regenerative design seeks not merely to lessen the harm of new development, but rather to make design and construction positive forces that repair natural and human systems.^28^ Figure 4-7 summarizes these positive impacts and contrasts them with more conventional approaches to design. One program that advances regenerative design principles is the Living Building Challenge, a voluntary certification framework with the goal of creating spaces that reconnect occupants with nature.^29^ A growing number of projects in the United States have received Living Building Challenge certification. The Omega Center for Sustainable Living in Rhinebeck, for example, was the first project in the world to achieve both this certification and a Leadership in Energy and Environmental Design (LEED) Platinum rating.^254^

**FIGURE 4-7:**
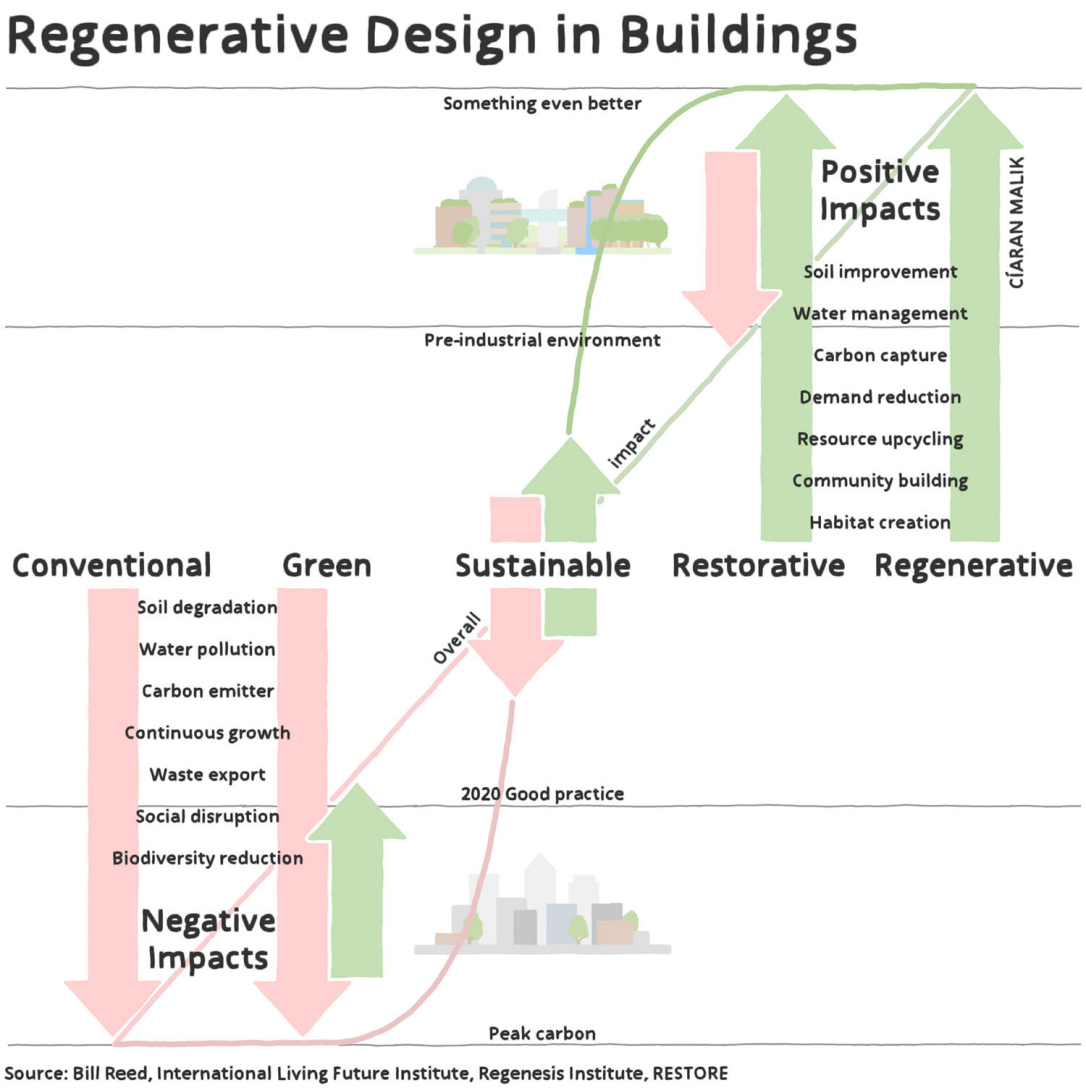
Regenerative design in buildings. Drawn by Cíaran Malik, Regenerative Practitioner.

Design features in “living buildings” build resilience to climate impacts. Energy efficiency coupled with on‐site renewable energy generation (e.g., solar) and energy storage can help protect building occupants and systems against grid power outages caused by climate events. Systems that capture and reuse all water used in buildings can help protect against water shortages or flooding caused by changing precipitation patterns. Features that restore, repair, and protect ecosystems can help mitigate the effects a changing climate has on the natural environment (e.g., wetland systems that reduce flood damage from extreme precipitation events).

Strategies intended to reduce greenhouse gas emissions from buildings also present opportunities to help buildings adapt to a changing climate. Some of the same features used to reduce energy use and emissions also help increase building resilience to climate impacts. For example, NYSERDA's Buildings of Excellence Competition funds multifamily projects that reduce energy consumption and per capita carbon emissions and improve occupant safety, health, and comfort while increasing building resiliency. The use of on‐site renewables and energy storage, for example, not only reduces emissions but also helps protect occupants from power outages caused by extreme weather events. Expansion of these types of programs and increased training within the industry can help reduce greenhouse gas emissions while also enhancing climate resilience in the built environment.

Building technology advancements also provide opportunities for both new construction and retrofits. Building management systems optimize building systems and performance, thereby lowering energy demand and reducing environmental impacts over the life of the building. In parallel, building performance simulation (BPS) advancements have the potential to advance resilience and sustainability in the coming decades.^255^ Smart microgrids have a similar impact on the environment and the added benefit of potentially increasing the resilience profiles of communities that generate their own power. Water harvesting reduces stormwater communitywide,^217^ and green roofs remove carbon from the atmosphere^256^ while the substrate of the planting medium adds insulation value to the roof, potentially reducing heating and cooling loads for the building.^257^

Buildings are physical infrastructure integrated within ecosystems to protect occupants from extreme weather and human‐made disasters. They should improve occupant health (or at the very least not adversely affect it) and reduce exposure to local pollutants.

### 6.2 Emerging topics and research needs

Many issues emerged during the development of this chapter of the assessment that could benefit from further investigation. Topics for additional research and policy development could include the following:

- **Retrofits**. Current retrofit efforts will need to be scaled up substantially to achieve broad‐ranging climate resilience; however, these efforts need to be achieved without exacerbating the housing affordability crisis. This is especially challenging in low‐ to moderate‐income multifamily retrofits. Retrofit technologies can be difficult to implement cost‐effectively and with minimal disruption to occupants. Future‐ready design should account for the expected useful life of the component parts of a building to assess the appropriate level of climate risk.^225^
- **Modeling**. BPS has been used to support high‐performance, energy‐efficient design for many years. In recent years, however, there has been increased interest and emerging research in using BPS to support resilient design as well. As an example, BPS can be used to assess and enhance passive survivability in both winter and during extreme heat events.^258^, ^259^ Such efforts can be applied at both the individual building level and the community level.^260^, ^261^, ^262^ However, more work is needed to better understand how buildings may perform in the future, such as quantifying the impact of different representation concentration pathways and incorporating future weather data.
- **Insurance**. Climate change–related impacts alter the insurability of buildings, resulting in very high premiums, or potentially no insurance options at all. For example, State Farm Insurance Companies recently halted the sale of all new home policies in California, citing wildfire risk.^263^ Changes like this will affect the buildings sector, which relies on property and casualty insurance to address risks. Higher‐risk areas could end up with insurance premiums that are unaffordable even for the wealthiest, let alone low‐ or middle‐income individuals.^264^ High‐value properties in flood‐prone areas could be affected by rising insurance rates, requiring hazard mitigation or shifts to self‐insurance for buildings.^265^
- **Climate‐related migration**. Climate‐related migration refers to the movement of people from areas more susceptible to climate change impacts to areas that are less so, for example, from coastal regions affected by tropical storms to inland regions.^266^ The Society and Economy chapter assesses what is known about migration in general, and it identifies several uncertainties. Climate‐related migration creates an emerging set of issues for buildings. Demand for shelter will increase in areas that are receiving climate migrants.^267^ Additionally, it is unknown where people might settle, either temporarily or permanently, in response to climate‐related displacement. In some cases, displaced people are migrating to urban areas.^268^ When thinking about climate migration, cities can be characterized in three ways: vulnerable cities that lose population and revenue; recipient cities that host climate migrants without preparation; and climate havens that are prepared and seek to be a destination for people leaving climate change–affected regions.^269^ Challenges particular to recipient cities include the temporary nature of the migration and implication for housing development; the tendency to build new housing in “greenfields” (i.e., previously undeveloped land) that are more exposed to climate risk than existing housing stock; informal settlements; and contribution to overcrowding and stresses on public services.^270^ Climate migration also has the potential to displace members of low‐income households in receiving cities.^269^

### 6.3 Conclusions

Buildings of all ages and functions are vulnerable to the impacts of climate change. The long lifespan of buildings, compounded with the expectation that climate change risks will grow over the next century, will result in an increased demand for future‐ready resilience strategies in new and existing buildings. Securing the next 100 years for New York State requires adapting and planning for tomorrow now.

Historically, the buildings sector's response to climate change has focused primarily on reducing carbon emissions. The future of buildings needs to involve not only reducing emissions but also adapting to changes in the climate and creating resilient buildings. Furthermore, climate justice strategies and targeted investments will be required to respond to the disproportionate impacts of climate change in overburdened communities.

New York State's buildings sector faces many challenges, but they provide an opportunity to proactively prepare for climate risks in ways that also enhance justice, support local economies, and result in healthier and safer communities.

Electrification and decarbonization are opportunities for improvements across the buildings sector. Implementing resilient new construction and retrofit projects is an opportunity to create a built environment that will continue to serve New York in decades to come. With some of the oldest building stock in the nation, the state can serve as an example of how to perform resilient electrification retrofits at scale. Electrification presents an opportunity to initiate additional adaptive and resilience measures, such as integrated photovoltaics, electric vehicles, and battery systems.

Transitioning to a resilient, decarbonized building stock in New York State will require a dedicated workforce.^271^ Workforce training efforts to meet this demand provide an opportunity to partner with overburdened communities to provide long‐term employment opportunities, support local economies, and transform the building stock.^272^, ^273^

For buildings to be responsive and adaptive to current and future conditions, there must be a continued and strategic engagement with—and connection to—regional planning and capital development processes. Understanding the interconnected and synergistic relationship between the regional “grid” and its relationship to the local “building” is ever more critical as hazards and risks continue to grow. Since buildings are interconnected to a complex web of energy, water, transportation, environmental, and other social systems,^145^ making improvements in the buildings sector provides cobenefits for these related sectors as well. Capital planning and improvement investment cannot solely be done at the individual building scale or through private investment alone. Investment at the community level enhances all systems across transportation, workforce, social systems, education, health, and housing sectors. Achieving a high resilience profile not only helps buildings resist the effects of hazards but also contributes to vital communities.

## 7 TRACEABLE ACCOUNTS

Traceable accounts examine each key finding in depth. They provide citations that support each assertion and present the authors’ assessment of confidence in each finding.

The assessment authors developed this chapter by reviewing journal articles, gray literature, building codes and standards, climate research reports, case studies, expert opinions, practitioner concerns, models and simulations, and the individual experiences of the chapter's Technical Workgroup members and Sector Advisors. The Technical Workgroup prioritized peer‐reviewed research in New York State, but also considered research from across the United States and international research. Depending on the availability of research, gray literature (e.g., technical reports) was also included. This chapter builds on findings from the *New York State Climate Hazards Profile* ^88^ and the 2019 *New York State Hazard Mitigation Plan* ^7^ with updated climate modeling estimates from Columbia University.^274^ The chapter also draws on information available through the New York State Division of Homeland Security and Emergency Services, National Oceanic and Atmospheric Administration, and FEMA websites.

### 7.1 Key Finding 1

**Buildings of all ages, functions, and locations across New York State are vulnerable to the impacts of climate change**. Each of the state's 12 assessment regions will experience a range of impacts, including more severe storms, coastal and inland flooding, and increasing temperatures, all of which can affect building structures and systems, operations, and occupants. Local and regional factors, including geography, zoning, and socioeconomic disparities, also affect vulnerability and will shape site‐specific adaptations.

#### 7.1.1 Description of evidence base

Climate hazards such as increased temperature, heat waves, changes in precipitation, sea level rise, pests, storms, and flooding impact buildings in a variety of ways.^42^, ^43^, ^44^, ^45^, ^61^, ^75^, ^81^, ^82^, ^88^, ^93^, ^94^, ^100^, ^121^, ^122^, ^123^, ^124^, ^127^, ^152^ Types of damage include structural damage to roofs, framing, foundation and walls; indoor building material damage; water damage and mold; damage to electrical, mechanical, and plumbing systems; and decreased building and system life spans.^7^, ^66^, ^68^, ^69^, ^81^, ^102^, ^103^, ^116^, ^118^, ^275^, ^276^, ^277^, ^278^, ^279^, ^280^, ^281^, ^282^, ^283^ Climate change also impacts the indoor environment through temperature; indoor air quality; water quality; and infiltration of infectious agents, pests, and toxins.^112^, ^119^, ^120^, ^284^

As sea levels rise and the frequency and intensity of storms increase, more buildings and their systems will be vulnerable to coastal and inland flooding.^7^, ^30^, ^64^, ^65^, ^80^ A hotter, wetter climate is conducive for pests to thrive and compromise building integrity.^3^, ^73^, ^121^, ^124^

Evidence is strong that site‐specific adaptation approaches will be more effective than more general ones.^204^

#### 7.1.2 New information and remaining uncertainties

Evidence related to regional differences in climate impacts to buildings is limited to inferences in regional variations due to hazard frequency and intensity, industry, and adaptive capacity. For example, convective storms and sea level rise are more likely to affect buildings on the North Atlantic coast, while pests and heat will have a disproportionate effect in regions dominated by agricultural buildings. Older post‐industrial cities will be at a higher risk for stronger and more frequent flooding due to aging infrastructure.^146^ However, there is a general lack of data on the current conditions and adaptive capacity of building stock across New York State. There is also uncertainty about how buildings might respond to hazards they have not yet been exposed to.

#### 7.1.3 Assessment of confidence based on evidence

Confidence is **very high** that all buildings in New York State are vulnerable to a variety of climate impacts. Confidence is also very high that the types of impacts to buildings, and therefore, effective resilience strategies, will vary across regions of the state and across building types.

### 7.2 Key Finding 2

**Given the long lifespan of buildings, new construction and retrofits that consider long‐term climate projections will better address future climate risk**. New York State's new and existing buildings are expected to support many generations of use, while individual components of those buildings may be replaced more frequently. As climate change risks increase throughout this century, design decisions that align buildings’ and building components’ life cycles with future climate projections and expected hazards will lead to more cost‐effective, sustainable, and future‐ready buildings.

#### 7.2.1 Description of evidence base

In New York State, 65% of buildings were built before 1970^285^ and 31% were built before 1940.^285^ Many of New York's buildings are currently in need of rehabilitation or repair; assessment of past damage suggests that vulnerability assessments and building code updates can help improve buildings’ climate resilience.^146^

Projected changes in the climate will continue to impact buildings into the future. Buildings built now will continue to be impacted by climate change in the mid‐ to late‐21st century.

Planning can help mitigate the impacts of climate hazards on buildings.^286^, ^287^, ^288^ Building science, operations, and systems design can also support resilience to chronic and acute impacts.^259^, ^262^, ^289^, ^290^, ^291^ Architecture and design fields also supply solutions to respond to hazards such as extreme heat.^211^, ^212^, ^220^, ^222^ Research indicates that adaptation efforts should be continual rather than one‐off events.^292^, ^293^

#### 7.2.2 New information and remaining uncertainties

There is uncertainty related to the frequency and magnitude of extreme weather patterns at regional level and at the building and site scales. For example, it is nearly impossible to predict the occurrence of compound events, yet research shows that such events frequently cause some of the most damaging impacts to buildings and building occupants. The severity of impacts to buildings is difficult to predict, and the consequences of impacts vary greatly by level of damage.

Regardless of this uncertainty, climate‐resilient buildings can also provide additional benefits. They are often more energy efficient, produce lower emissions, and provide greater comfort and indoor air quality. While adaptation does not always have a clear and certain short‐term return on investment,^294^ builders, developers, and planners can seek retrofits or strategies that serve both climate hazard resilience and energy efficiency/decarbonization goals. To date, the buildings sector has prioritized mitigating negative environmental impacts and improving energy efficiency rather than promoting hazard mitigation and resilience.^295^ Energy efficiency and carbon reduction projects are both opportunities for building climate resilience,^205^ though these are not typically linked in funding strategies.^156^

#### 7.2.3 Assessment of confidence based on evidence

Confidence is **very high** that climate change risks and impacts will increase during the life span of new and retrofitted buildings. For buildings designed or retrofitted with forward‐looking construction techniques, confidence is very high that these strategies will enhance the buildings’ ability to withstand future climate impacts.

### 7.3 Key Finding 3

**Climate impacts to buildings can ripple to many different parts of a community**. Buildings are integrated and interdependent with other sectors, including agriculture, energy, transportation, and health services. Damage to buildings can disrupt these interdependent systems and compound the direct impacts of building loss and harm to individuals and communities. Addressing these cross‐sector impacts will require not only resilient building design but also a multidisciplinary approach and insight into interconnected sectors to improve community resilience.

#### 7.3.1 Description of evidence base

Buildings provide humans with their primary protection from exposure to weather‐ and climate‐related hazards. Ample research documents the impact on building occupants when buildings and building systems are compromised or destroyed. These impacts include heat‐ and cold‐related exposure or death; respiratory illness from exposure to mold, allergens, poor outdoor air quality and toxins; waterborne disease; chemical and toxin exposure through carbon monoxide, pesticides, chemical release, and outdoor air exposure; psychological distress; financial impacts to household budgets; loss of jobs or places of business; and displacement from homes.^39^, ^53^, ^73^, ^85^, ^86^, ^115^, ^126^, ^138^, ^296^, ^297^, ^298^, ^299^, ^300^ Impacts to buildings and building occupants further impact communities by damaging essential facilities such as schools, community centers, and hospitals; disrupting communities’ economies and food, education and health care systems; disrupting communities’ social fabrics; and affecting the financial health of local governments through loss of tax base and because of direct expenditures from and opportunity costs of focusing on disaster response.^135^, ^139^, ^143^, ^301^

Buildings are interdependent with the surrounding environment. Research shows how adaptations such as green roofs can lower heating costs and improve public health outcomes for residents while also reducing the heat island effect in the surrounding neighborhood.^217^ Living buildings can benefit the biodiversity and environmental health of the surrounding neighborhood or area.

Buildings such as schools, hospitals, and medical facilities play an important role in emergency response.^302^ Damage to agricultural buildings and public service facilities makes communities and larger systems more vulnerable. Compound events can further compromise community wellbeing.^303^ When critical buildings are made more resilient, the entire community benefits, not just the buildings’ immediate occupants and users.

#### 7.3.2 New information and remaining uncertainties

The need for multidisciplinary approaches to address the links between climate change, buildings, and community health and wellbeing is well documented.^24^, ^39^, ^304^ There is a lack of evidence, however, regarding successful mechanisms for collaboration across sectors. Planning can help mitigate the impacts of climate hazards on buildings.^286^, ^287^ Strategies to prevent building damage during hazards come from structural, civil, and environmental engineering.^305^ Building science, operations, and systems design can also support resilience to chronic and acute impacts.^259^, ^262^, ^289^, ^290^, ^291^ Architecture and design fields also supply solutions to respond to hazards such as extreme heat.^211^, ^212^, ^220^, ^222^ Pest management is needed to respond to the spread of pests and rodent and insect‐borne diseases due to climate change.^121^, ^122^, ^123^ Lastly, emergency management is needed during and after disasters.^234^, ^235^, ^306^, ^307^

#### 7.3.3 Assessment of confidence based on evidence

There is **very high** confidence that buildings moderate exposure to climate impacts by providing protection from the environment. Confidence is also **very high** that buildings are integrated and interdependent on other sectors. This interdependence can compound the impacts of a climate hazard. Confidence is **medium** that addressing these impacts will require a multidisciplinary approach and diverse perspectives.

### 7.4 Key Finding 4

**Communities of color, Tribal communities, and low‐income communities are more likely to congregate, live, and work in buildings that have greater exposure to climate hazards**. In addition, people who are very young or very old, as well as those experiencing physical or developmental disabilities, are more vulnerable to building failures. Additional resources and policies will be required to respond to the disproportionate impacts of climate change in these communities.

#### 7.4.1 Description of evidence base

Frontline communities are impacted more immediately by climate change, either due to their vulnerability or their exposure.^160^, ^308^ Climate change disproportionately affects the rental housing market, low‐income renters, households of color, and people who speak English as a second language.^143^ Renters and people living in subsidized housing are more vulnerable to displacement after storms.^163^ Some buildings, such as schools, manufactured homes, prisons, and nursing homes, support or house more vulnerable populations. Impacts to communities from climate hazards are compounded due to the history of residential segregation, disinvestment, and proximity to industrial and aging infrastructure. Housing developed for low‐income residents is often located in more environmentally at‐risk areas because the land is less expensive.^13^, ^19^, ^124^ Vulnerable populations and associated structures are often located in flood zones.^161^, ^162^

One approach to addressing the intersectionality of climate and housing justice is through the “just transition” framework put forth in the Climate Leadership and Community Protection Act scoping plan.^309^

#### 7.4.2 New information and remaining uncertainties

There is uncertainty related to how vulnerable populations will be impacted by climate migration. Migration dynamics may exacerbate existing inequities. For example, low‐income communities may not have the resources to leave areas at higher risk from climate impacts. Equitable resilience will depend on the effectiveness of public policies and funding allocations.

#### 7.4.3 Assessment of confidence based on evidence

Confidence is **high** that communities of color, Tribal communities, and low‐income communities are more likely to congregate, live, and work in buildings that have a greater exposure to climate hazards due to systemic inequities. Confidence is **very high** that additional funding and policies will be required to respond to the disproportionate impacts of climate change in these communities.

### 7.5 Key Finding 5

**Individual adaptation and resilience strategies can address multiple climate impacts**. Buildings in New York State will face various climate hazards over their useful lives. Strategies such as green roofs, for example, can address both flooding and the urban heat island effect, while also providing cobenefits like reducing cooling load. Resilient design strategies can be implemented in many types of buildings and can increase community‐level resilience to climate change.

#### 7.5.1 Description of evidence base

Though different buildings experience different vulnerabilities, resilience measures are often similar. Research supports the use of green infrastructure that mitigates multiple hazards, including extreme heat and flooding.^241^, ^244^ Strengthening building systems and improving the grid can have benefits across climate‐related extreme weather events.^310^

Research supports the use of building codes to promote resilience.^311^ Local governments such as New York City are authorized by the state to adopt both uniform and energy codes that are more stringent than the state codes. However, the positive effects of code improvements are slow to accumulate; most buildings in the state will remain untouched for several decades.^312^ Standards are less effective at protecting communities from the effects of climate‐related disasters.

Because of the ongoing cost of building operations and maintenance, there are more funding opportunities for climate resilience than in other sectors due to the cost savings that come from building retrofits. Though gaps remain, there are a variety of funding mechanisms that vary by building type and industry and across scales of development.^313^

#### 7.5.2 New information and remaining uncertainties

There remains a lack of information about how to optimize buildings for multiple climate hazards simultaneously. There are limited real‐world examples of effective resilient retrofits. Without feedback on performance, it is difficult to prioritize adaptation approaches. Building for resilience and redundancy may compromise efficiency. An analysis of these tradeoffs would help building designers, owners, and operators set priorities to optimize for resilient outcomes.

#### 7.5.3 Assessment of confidence based on evidence

There is **very high** confidence that adaptation and resilience strategies can address multiple impacts at the same time. Confidence is also **very high** that codes and standards can encourage resilient building design.

## AUTHOR CONTRIBUTIONS

N.B.R.: Drafting, revising, and editing the manuscript; manuscript compilation and review; cochair of the Buildings Technical Workgroup. C.B.: Drafting, revising, and editing the manuscript; manuscript compilation and review; cochair of the Buildings Technical Workgroup. I.A.: Drafting, revising, and editing the manuscript. E.B.: Drafting, revising, and editing the manuscript. S.C.: Drafting, revising, and editing the manuscript. J.E.: Drafting, revising, and editing the manuscript. B.G.: Drafting, revising, and editing the manuscript. M.T.H.: Drafting, revising, and editing the manuscript. J.L.: Drafting, revising, and editing the manuscript. O.O.: Drafting, revising, and editing the manuscript. L.S.: Drafting, revising, and editing the manuscript.

## COMPETING INTERESTS

The authors declare no competing interests.

### PEER REVIEW

The peer review history for this article is available at: https://publons.com/publon/10.1111/nyas.15200
